# Acox2 deficiency induces cardiac dysfunction via cross-organ metabolic reprogramming in mice

**DOI:** 10.1186/s12967-026-08797-5

**Published:** 2026-08-10

**Authors:** Cancan Yao, Yan Chen, Zhouping Lu, Ke Cai, Feifei Wang, Boxuan Wu, Muling Zeng, Eudald Casals, Yuan Zhang, Xiangyu Zhou

**Affiliations:** 1 Obstetrics and Gynecology Hospital of Fudan University, Shanghai Key Lab of Reproduction and Development, Shanghai Key Lab of Female Reproductive Endocrine Related Diseases, Shanghai, 200090 China; 2 Shanghai Key Laboratory of Maternal Fetal Medicine, Department of Assisted Reproduction, Shanghai First Maternity and Infant Hospital, Tongji University School of Medicine, Shanghai, 201204 China; 3 Institute For Developmental and Regenerative Cardiovascular Medicine, MOE-Shanghai Key Laboratory of Children’s Environmental Health, Xinhua Hospital, Shanghai Jiao Tong University School of Medicine, Shanghai, China; 4 Institut de Ciència de Materials de Barcelona, ICMAB (CSIC), Campus UAB, Bellaterra, 08193 Spain; 5 Vall d’Hebron Institut de Recerca (VHIR), Barcelona, 08035 Spain; 6 Premium Research SL, Guadalajara, 19003 Spain; 7 Obstetrics and Gynecology Hospital of Fudan University, 128 Shenyang Road, Shanghai, 200090 China

**Keywords:** ACOX2, Succinylation, Secondary cardiomyopathy, Heart failure, Hepato-cardial syndrome, Cross-organ

## Abstract

**Background:**

Secondary cardiomyopathies are a major cause of heart failure (HF) with diverse etiologies and high mortality rates. For example, over 50% of patients with end-stage liver disease develop cardiac dysfunction, clinically termed cirrhotic cardiomyopathy (CCM). While serum metabolites mediating cross-organ communication are known to play a critical role in CCM development, the underlying pathogenic mechanisms remain poorly understood due to a lack of optimal experimental models.

**Methods:**

We generated Acox2 knockout (KO) mice using CRISPR/Cas9 technology to investigate the role of ACOX2, a rate-limiting peroxisomal enzyme. Cardiac function was assessed via echocardiography. We performed RNA sequencing and quantitative proteomic analyses to evaluate cardiac gene expression. Furthermore, global succinylome profiling was conducted to determine changes in protein succinylation, and global metabolomics analysis was used to identify significantly differentially expressed metabolites (SDMs).

**Results:**

Acox2 KO mice successfully recapitulated the hepatic phenotypes observed in patients with ACOX2 homozygous mutations, including liver fibrosis and lymphocytic infiltration. Given that ACOX2 is not expressed in the mouse heart throughout its lifespan, we initially hypothesized that Acox2 deficiency would have no direct impact on cardiac function. However, by 6 months of age, Acox2 KO mice exhibited significantly compromised cardiac parameters via echocardiography, alongside elevated biomarkers of cardiomyopathy and heart failure. While Acox2 deficiency had limited impact on the cardiac proteome, global succinylome profiling revealed a pervasive decrease in protein succinylation in Acox2 KO hearts. Notably, approximately 60% of these hypo-succinylated sites were located on mitochondrial and myofibrillar proteins, including SUCLA2 (K94), CS (K321), FH (K470), MDH1 (K110), OGDH (K999), TNNC1 (K86), VCL (K453), MYH7 (K83), and MYL3 (K9)—a profile tightly associated with cardiomyopathy. Seahorse assays and scanning electron microscopy further indicated impaired mitochondrial function in Acox2 KO mice. Metabolomic analysis revealed a dramatic accumulation of plasma C27 bile acid intermediates and a concomitant decrease in cardiac succinyl-CoA levels.

**Conclusions:**

Our findings underscore the critical role of the succinylation landscape in maintaining cardiac function. The cardiac pathology observed in Acox2 KO mice originates from cross-organ metabolic signaling, providing new insights into the pathogenesis of secondary cardiomyopathies, such as cirrhotic cardiomyopathy.

**Supplementary Information:**

The online version contains supplementary material available at https://doi.org/10.1186/s12967-026-08797-5.

## Background

Cardiomyopathy is a leading cause of non-ischemic heart failure (HF) [1, 2]. Despite significant advances in HF management, mortality rates associated with cardiomyopathy remain unacceptably high [3]. Cardiomyopathies are broadly classified as either primary or secondary [4]. Cirrhotic cardiomyopathy (CCM), a classic manifestation of secondary cardiomyopathy, arises as a complication of liver dysfunction and is often categorized as a hepato-cardiac syndrome [5]. CCM is characterized by cardiac dysfunction, including impaired systolic and diastolic contractility, as well as electrophysiological abnormalities, such as QT interval prolongation [6]. This condition affects over 50% of patients with end-stage liver disease [7]. Notably, cardiac dysfunction in patients with CCM can be reversed following liver transplantation [8]. Despite its clinical significance, progress in the diagnosis and treatment of CCM remains stagnant, largely due to a lack of efficient experimental models capable of elucidating its underlying pathogenic mechanisms.

Cross-organ metabolic reprogramming, mediated by circulating serum metabolites, is currently considered the primary pathogenesis of CCM. Specifically, various acyl-CoAs—including acetyl-CoA, succinyl-CoA, and crotonyl-CoA—serve as essential donors for corresponding post-translational modifications (PTMs) [9, 10]. By adding or removing chemical groups, PTMs can directly modulate protein properties, acting as “molecular switches” that regulate metabolic enzyme activity and trigger subsequent metabolic reprogramming. For instance, lysine succinylation involves the transfer of a negatively charged succinyl group to a lysine residue via succinyl-CoA [11]. Recent research has increasingly highlighted the critical role of succinylation in cardiac homeostasis [12–14]. Notably, a reduction in the succinylation levels of myofibrillar and mitochondrial proteins has been documented in patients with ischemic cardiomyopathy and chronic HF [15, 16], underscoring the vital contribution of this PTM to the maintenance of cardiac function.

*Acox2* belongs to the acyl-CoA oxidase family and encodes a rate-limiting enzyme that is critical for two key metabolic pathways within peroxisomes: the synthesis of bile acids—via the shortening of C27 cholesterol derivatives—and the degradation of branched-chain fatty acids [17, 18]. The high evolutionary conservation of Acox2, spanning from bacteria to plant species such as Arabidopsis thaliana and tomato, underscores its fundamental metabolic importance [19]. Humans harboring homozygous ACOX2 mutations (e.g., p.Y69* and p.R225W) present with accumulated C27 intermediates, elevated transaminases and liver fibrosis during childhood [20, 21]. To investigate these conditions, we previously generated an Acox2 knockout (KO) mouse model that successfully recapitulated the hepatic phenotypes observed in patients with ACOX2 mutations [22, 23]. Intriguingly, ACOX2 deficiency has also been implicated in cardiac metabolic shifts in patients presenting with primary malignant cardiac tumors [24, 25].

Our research specifically focuses on Acox2 because it is naturally absent from the mouse heart throughout the entire lifespan, as indicated by the Expression Atlas database [26]; this renders it an optimal model for distinguishing secondary cardiomyopathy driven by non-cardiac influences. Here, we report that Acox2 KO mice develop the characteristic phenotypes of dilated cardiomyopathy (DCM) and heart failure. Notably, succinylation levels on non-histone proteins were downregulated in the hearts of Acox2 KO mice. Given the absence of Acox2 expression in the murine heart, it is highly plausible that hepato-cardiac crosstalk, via metabolic reprogramming, contributes to the cardiac pathology observed in Acox2 KO mice.

## Methods

### Animal models

Generation of Acox2-KO mice models were supported by Gempharmatech Co., Ltd. (Nanjing, China) using the CRISPR-Cas9 technique, as previously described [23]. Briefly, fertilized embryos (zygotes) were collected from their oviducts. Cas9 mRNA (100 ng/µL) and sgRNA (50 ng/µL) were mixed and injected into the cytoplasm of fertilized eggs with both pronuclei visible in CZB (Chatot–Ziomek–Bavister) medium. The injected zygotes were then cultured in Quinn’s Advantage cleavage medium (In-Vitro Fertilization, Inc.) containing SCR7 (50 μm, TOCRIS) for about 24 h, and 18–20 2-cell stage embryos were transferred into the oviduct of a pseudo-pregnant ICR female mouse at 0.5 dpc. All mice had access to food and water. All experiments were performed in accordance with Health Guide for the Care and Use of Laboratory Animals. Genomic DNA was extracted from tail tips to perform PCR-based genotyping. Homozygous mutant mice (derived from heterozygous parents) and wild-type (WT) littermates were used in all studies.

### Echocardiography

Transthoracic echocardiography was performed in mice using a Vevo3100 and 2100 system (FUJIFILM VisualSonics Inc.) with an MS400 transducer following anaesthesia with 2% isoflurane gas and 0.8 L/min oxygen. Echocardiography was used to assess left ventricle (LV) systolic and diastolic functions with a 2-dimensional guided M-mode ultrasound system (Acuson Sequoia, Siemens Medical Solutions, Mountain View, CA, USA). Two-dimensional images from a short-axis view of the LV were obtained at the level of the papillary muscles using a 15 MHz linear transducer at a paper speed of 100 mm/s. Echocardiography was performed before opening the chest (baseline reading) and during the course of the study for all groups. Three consecutive cardiac cycles were analysed, and the average values were used for data analysis.

### Histopathology staining

Heart, liver and kidney tissues were fixed in 4% paraformaldehyde, embedded in paraffin, cut into 5 μm-thick sections, and placed on glass slides. Tissue sections were subjected to haematoxylin and eosin, Sirius Red, or Masson’s trichrome staining according to standard procedures. For Sirius Red staining, fibrosis was visualised using a Picrosirius Red Staining Kit (Polysciences, Inc.). Fibrosis was visualised using a Masson’s Trichrome Staining Kit (Polysciences, Inc.).

### Immunofluorescence staining

Paraffin-embedded heart tissue sections were rehydrated, and antigens were retrieved using heat in the presence of citrate. The sections were then blocked with 10% goat serum and incubated overnight at 4°C with primary mouse anti-atrial natriuretic peptide (Anp) or anti-cTnT antibodies. On the following day, the sections were washed in 1× Tris-buffered saline and 0.1% Tween-20 detergent and incubated with secondary Alexa Fluor 488-conjugated goat anti-mouse antibodies (Invitrogen, A-11001). 4’,6-diamidino-2-phenylindole was subsequently added for nuclear staining. The slides were observed under a fluorescence microscope, and the images were obtained in multitracking mode using a confocal laser scanning microscope (LSM880-Airyscan; Carl Zeiss).

### RNA isolation and library preparation

Total RNA was extracted using the TRIzol reagent (Invitrogen, CA, USA). RNA purity and quantification were evaluated using the NanoDrop 2000 spectrophotometer (Thermo Scientific, USA). RNA integrity was assessed using the Agilent 2100 Bioanalyzer (Agilent Technologies, Santa Clara, CA, USA). Then the libraries were constructed using VAHTS Universal V6 RNA-seq Library Prep Kit according to the manufacturer’s instructions.

### RNA sequencing analysis

The libraries were sequenced on an Illumina Novaseq 6000 platform and 150 bp paired-end readswere generated. Raw reads of fastq format were firstly processed using fastp1.0 and the low quality reads were removed to obtain the clean reads. The clean reads were mapped to the reference genome using HISAT2. Fragments per kilobase of exon per million mapped reads (FPKM) of each gene was calculated and the read counts of each gene were obtained by HTSeq-count. Differential expression analysis was performed using the DESeq2. Q-value < 0.05 and fold-change > 1.5 was set as the threshold for significantly differential expression gene (DEGs).

### Quantitative real-time PCR

cDNA synthesis was performed using the HiScript III All-in-one RT SuperMix Perfect for qPCR (Vazyme, Nanjing, China). Quantitative real-time PCR (qRT-PCR) was subsequently conducted on an Applied Biosystems StepOnePlus instrument (Thermo Fisher Scientific, Waltham, MA, USA) using the 2× SYBR Green qPCR Master Mix (Vazyme) according to the manufacturer’s instructions. The relative mRNA expression levels were calculated using the 2(−ΔΔCT) method. The primer used are as follows: Acox2-F:5’-CCTTCCTAGACCTGCTTCCC-3’, Acox2-R: 5’-TGTCCGTCATAA CAGCCAAG-3’; Actin-F: 5’-GGCTGTATTCCCCTCCATCG-3’, Actin-R: 5’-CCA GTTGGTAACAATGCCATGT-3’.

### Enrichment of succinylated peptides

To enrich for Suc-modified peptides, tryptic peptides were dissolved in NETN buffer (100 mM NaCl, 1 mM EDTA, 50 mM Tris-HCl, and 0.5% NP-40 [pH 8.0]) and then incubated overnight at 4 °C with antibody-conjugated beads (PTM-402, PTM Bio, Inc., Hangzhou) at a ratio of 15 µL beads/mg protein. The antibody-conjugated beads were washed four times with NETN buffer and twice with ddH2O. Succinylated peptides were eluted with elution buffer containing 0.1% trifluoroacetic acid. For LC-MS/MS analysis, the resulting peptides were desalted using C18 ZipTips (Millipore), according to the manufacturer’s instructions.

### LC-MS/MS analysis

Tryptic peptides were dissolved in solvent A (0.1% formic acid and 2% acetonitrile in water) and directly loaded onto a homemade reverse-phase analytical column (25 cm length, 75/100 µm i.d.). Peptides were separated using a nanoElute UHPLC system (Bruker Daltonics) and a gradient of solvent B (0.1% formic acid in acetonitrile). The solvent B concentration increased from 6% to 24% in 70 min, from 24% to 35% in 14 min, and then up to 80% in 3 min. The solvent B concentration was held at 80% for the last 3 min. A constant flow rate of 450 nL/min was used. The peptides were subjected to capillary source analysis, followed by analysis with a timeTOF Pro (Bruker Daltonics) mass spectrometer (MS). The applied electrospray voltage was 1.60 kV. The precursor and fragment ions were analysed using a TOF detector with an MS/MS scan range of 100–1700 m/z. The timsTOF Pro instrument was operated in the parallel accumulation serial fragmentation (PASEF) mode. Precursor ions with charge states of 0–5 were selected for fragmentation, and 10 PASEF-MS/MS scans were acquired per cycle. The dynamic-exclusion feature was set at 30 s.

### GO and KEGG enrichment analyses

We obtained an annotated Gene Ontology (GO) proteome from the UniProt-GOA Database (http://www.ebi.ac.uk/GOA/). Proteins were classified by GO annotation into three categories: biological processes, cellular components, and molecular functions. For each category, a two-tailed Fisher’s exact test was used to test for the enrichment of differentially expressed proteins (DEPs) against all identified proteins. GO terms with a p value of < 0.05 were considered to be significantly enriched.

We used an online Kyoto Encyclopedia of Genes and Genomes (KEGG) service tool (KAAS) to annotate the protein descriptions found in the KEGG database. BLAST alignments (Blastp, E-value < 1e − 4) were performed with the identified proteins. The highest BLAST-alignment score of each alignment was selected for annotation. Pathways with corrected p-values of < 0.05 were considered to be significantly enriched. These pathways were classified into hierarchical categories according to the KEGG website.

### Isolation of primary mouse cardiomyocytes

Extraction of primary adult mouse cardiomyocytes was support by Shanghai Yanlu Biomedical Co., Ltd. Briefly, Mice are administered an intraperitoneal injection of 2.5 U/g heparin for anticoagulation and incubated for 30 min. Following this, they are euthanized by cervical dislocation. The thoracic cavity is opened, and the chest is fixed with sutures. The aorta is then severed, and the heart tissue is rapidly excised and placed on a Langendorff isolated heart perfusion system (pre-warmed to 37 °C). The heart is initially perfused with a buffer at 3 mL/min for 5 min, followed by perfusion with a digestion solution for 12–15 min, or until the heart enlarges and appears white and translucent, at which point perfusion is stopped. The heart is then cut below the atria and placed into 5 mL of digestion solution. The heart is gently disrupted using blunt tearing and trituration. A stop solution is added to terminate digestion. The cell suspension is filtered through a 100 µM mesh sieve. After allowing the cells to settle at room temperature for 15 min, the supernatant is discarded. This supernatant removal process is repeated twice. The cells are then sequentially resuspended in solutions containing 0.1, 0.3, 0.6, and 1.0 mmol/L Ca²⁺ for 10 min each to allow for re-equilibration of calcium. The purity of the cardiomyocytes is assessed by immunofluorescence staining for cardiac markers such as α-Actinin and cTnT, which are used for subsequent functional analysis including Seahorse and JC-1 staining.

### Seahorse assay

The Seahorse XFe96 Analyzer served as the apparatus for quantifying oxygen consumption rates (OCRs) in primary CMs, as elucidated in previous documentation. Cells were seeded at a density of 8000 cells per well on XFe96 Seahorse plates (Agilent) precoated with 0.01% poly-L-lysine. After 2 h of culture, OCR measurements were conducted using a Seahorse Bioscience XFe96 Extracellular Flux Analyzer. The assay medium was concocted by supplementing XF Assay Medium (Seahorse Bioscience) with 10 mM glucose, 2 mM L-glutamine, and 1 mM sodium pyruvate (pH 7.4). After acquiring baseline measurements, the mitochondrial stress test profile was delineated through sequential injections of 1.5 µM oligomycin (ATP synthase inhibitor), 3 µM FCCP (mitochondrial uncoupler), and a combination of 1 µM antimycin A (complex III inhibitor) and 1 µM rotenone (complex I inhibitor). Computational analyses adhered to the procedures outlined in the Seahorse manual and XF Seahorse Mito Stress Test kit user guide.

### JC-1 staining

Primary cardiomyocytes were resuspended in 1 mL of JC-1 working solution and incubated in a 37 °C, 5% CO₂ incubator for 25 min. Subsequently, the cells were seeded into confocal dishes and washed twice with phosphate-buffered saline (PBS). Observations were performed under a laser scanning confocal microscope with an excitation wavelength of 488 nm. Fluorescence emissions were collected at 590 nm (red fluorescence) and 530 nm (green fluorescence).

### Scanning electron microscopy (SEM)

Heart tissue from C57 mice was harvested, cut into 2 mm fragments, and thereafter fixed in 2.5% glutaraldehyde and 4% paraformaldehyde in 0.1 M PBS, pH 7.4 at room temperature for 24 h. This was followed by treatment with osmium tetroxide for 3 h and dehydrated in a series of graded alcohol, infiltrated and embedded in Araldite resin, and allowed for polymerization at 80 °C for 72 h. Ultrathin (60 nm) sections were made in Leica Ultra cut UCT-GA-D/E-1/00, mounted on copper grids, and stained with Uranyl acetate. Imaging was performed on the JEOLJEM2100 TEM.

### Serum untargeted LC-MS/MS analysis

Metabolomic data analysis was supported by Shanghai OE Biotech Co., Ltd. (Shanghai, China). Metabolic profiling was conducted using an ACQUITY UPLC I-Class plus system (Waters Corporation, Milford, USA) coupled with a Q-Exactive mass spectrometer equipped with a heated electrospray ionization (ESI) source (Thermo Fisher Scientific, Waltham, MA, USA). Chromatographic separation was achieved on an ACQUITY UPLC HSS T3 column (1.8 μm, 2.1 × 100 mm) in both positive and negative ion modes. The mobile phase consisted of (A) water (containing 0.1% formic acid, v/v) and (B) acetonitrile. The gradient elution program was as follows: 0–2 min, 5% B; 4 min, 30% B; 8 min, 50% B; 10 min, 80% B; 14–15 min, 100% B; 15.1–16 min, 5% B. The flow rate was 0.35 mL/min, and the column temperature was maintained at 45 °C. Samples were kept at 10 °C during the analysis. The mass range was set from m/z 70 to 1050. The resolution was set to 60,000 for full MS scans and 15,000 for HCD MS/MS scans. The collision energies were 10, 20, and 40 eV. The mass spectrometer parameters were as follows: spray voltage, 3,800 V (+) and 3,200 V (−); sheath gas flow rate, 35 arbitrary units; auxiliary gas flow rate, 8 arbitrary units; capillary temperature, 320 °C; auxiliary gas heater temperature, 350 °C; and S-lens RF level, 50.

### Succinate measurement by KIT

The Succinate Colorimetric Assay Kit (Sigma, MAK184) was used for determining succinate levels in mice tissues. Briefly, fresh tissue sample (10 mg) was rapidly homogenized on ice in 100 m L of ice-cold Succinate Assay Buffer. Centrifuge at 10,000 g for 5 min to remove insoluble material. Collect the supernatant and mix with 50 uL of the Reaction Mix in each well. Incubate the reaction for 30 min and measure the absorbance at 450 nm (A450). Subtract the Sample Blank value from the sample reading to obtain the corrected measurement. Using the corrected measurement, the amount of Succinate present in the sample was determined from the standard curve.

### Succinyl-CoA and succinic acid measurement by LC/MS

The identification and quantification of metabolites were performed using LC–MS/MS in multiple reaction monitoring (MRM) mode. For in vivo succinyl-CoA (Suc-CoA) measurement, heart tissues (50 mg) were collected and homogenized in 1 mL of pre-cooled 80% methanol. Samples were lysed on a rotating shaker at − 20 °C overnight, followed by centrifugation at 12 000 × g for 30 min at 4 °C. For serum Suc-CoA and succinic acid measurement, 60 µL of serum was mixed with 450 µL of 80% methanol, vortexed for 10 s, and centrifuged at 12 000 × g for 10 min at 4 °C. The supernatant was collected, evaporated, resuspended, and analyzed by LC-MS.

Samples (10 µL) were injected into a SHIMADZU LC-30AB LC system coupled to a QTRAP 7500 mass spectrometer (SCIEX). Chromatographic separation was performed using a HILIC BEH column (1.7 μm, 2.1 mm × 100 mm; Waters). Mobile phase A consisted of 10 mm ammonium formate in water with 0.2% ammonia; mobile phase B was 100% acetonitrile. The flow rate was 0.2 mL min − 1 with a 15 min gradient: 100% phase A for 1 min, a linear increase to 90% phase B over 5 min, holding at 90% phase B for 2 min, returning to 0% phase B over 0.1 min, and holding at 100% phase A for 6.9 min. The system was operated in negative ionization mode. The specific transition monitored for Suc-CoA was m/z 865.7492 → 407.8342. MRM data were processed using Skyline software.

### Site-directed mutagenesis

pEnter-MYL3-Flag-His (NM_000258), pEnter-VCL-Flag-His (NM_003373) and pEnter-TNNC1-Flag-His (NM_003280) ORF clones were obtained from WZ Biosciences, Inc. (Jinan, China). A QuickChange SiteDirected Mutagenesis Kit (Stratagene, Agilent Technologies) was used to modify the Lysine(K) Suc sites at MYL3, VCL and TNNC1 to encode R and generate the mutant MYL3 (K4R and K9R), VCL(K453R), TNNC1(K86R) variants, which was confirmed by Sanger sequencing.

### Immunoprecipitation assay

HEK-293T and H9C2 cells were grown in DMEM High Glucose medium (4.5 g glucose/L) (Gibco), supplemented with 10% FBS (Gibco) and 100 IU/mL penicillin in a 5% CO2 atmosphere. Then, the cells were transfected with expression constructs encoding Flag-tagged recombinant proteins using Lipofectamine 3000 (L3000-008, Invitrogen). After 48 h, cells were collected with 500 µl of IP lysis buffer (Thermo Fisher Scientific). Add 40 µl of DYKDDDDK-Nanoab-Magneticbeads (LabLead) to the 500 µg of cell lysate, and rotate and mix at 4 °C for 30 min to 1 h for binding. Separate the magnetic beads on a magnetic rack and discard the supernatant. Resuspend the beads in 30 µl of 2×SDS-sample buffer. Heat at 95 °C for 10 min to fully denature, and perform SDS-PAGE on the collected products.

### Immunoblotting

Proteins were extracted using RIPA lysis buffer containing protease inhibitors (NCM biotech). Next, the specimens underwent a brief sonication process, and the resultant supernatant was meticulously collected following centrifugation at 12,000 rpm for 15 min at 4 °C. Quantification of all protein samples transpired using the BCA kit (Beyotime). After SDS-PAGE, the proteins underwent transference onto 0.45 μm PVDF membranes (EMD Millipore). These membranes were then subjected to block in 5% skim milk for 2 h at room temperature and then probed with one of the following primary antibodies in either 5% non-fat milk or 5% bovine serum albumin: Acox2 (santa cruz, sc-514320), anti-pan K crotonylation (PTM, 501, 502), anti-pan K acetylation (PTM, 101), anti-pan K Suc (PTM, 401, 402), anti-pan ubiquitin (PTM, 1107), anti-ANP (Santa Cruz, sc-515701), anti-β-actin (CST, 4970), anti-GAPDH (CST, 8844),anti-Flag (CST, 8146, 14793), anti-beta-Tubulin (CST, 2146), α-SMA (CST, 192457), Ccn2(Santa Cruz, sc-373936), MYH7 (abmart, 756635S), Anp (santa cruz, sc515701), Bnp (abmart, PS00154S), COL3A1(abcam, ab184993). After 3 meticulous washes with TBST, the membranes received further incubation with the appropriate secondary antibody for 1 h at room temperature, concluding with visualization through the ECL ChemiDoc XRS+ system.

### Statistical analysis

Data are expressed as the mean ± standard error of the mean (SEM) for individual samples. The number of biological replicates (n) is specified in the respective figure legends. Statistical significance between two groups was determined using a two-tailed unpaired Student’s t-test, while comparisons among three or more groups were performed using one-way analysis of variance (ANOVA) followed by appropriate post-hoc tests. All analyses were conducted using GraphPad Prism 5.01 (GraphPad Software, Inc., La Jolla, CA, USA). A p-value of < 0.05 was considered statistically significant. For transcriptome, a q-value < 0.05 was used as the threshold for identifying differentially expressed genes (DEGs). For metabolome, both p-values and q-values were calculated, with *p* < 0.05 set as the criterion for identifying significantly altered metabolites for downstream enrichment analysis. For proteome and succinylome analyses, a p-value < 0.05 was used to define significantly differentially expressed proteins.

## Results

### ACOX2 is not expressed in the murine heart from the embryonic stage to adulthood

**Fig. 1:**
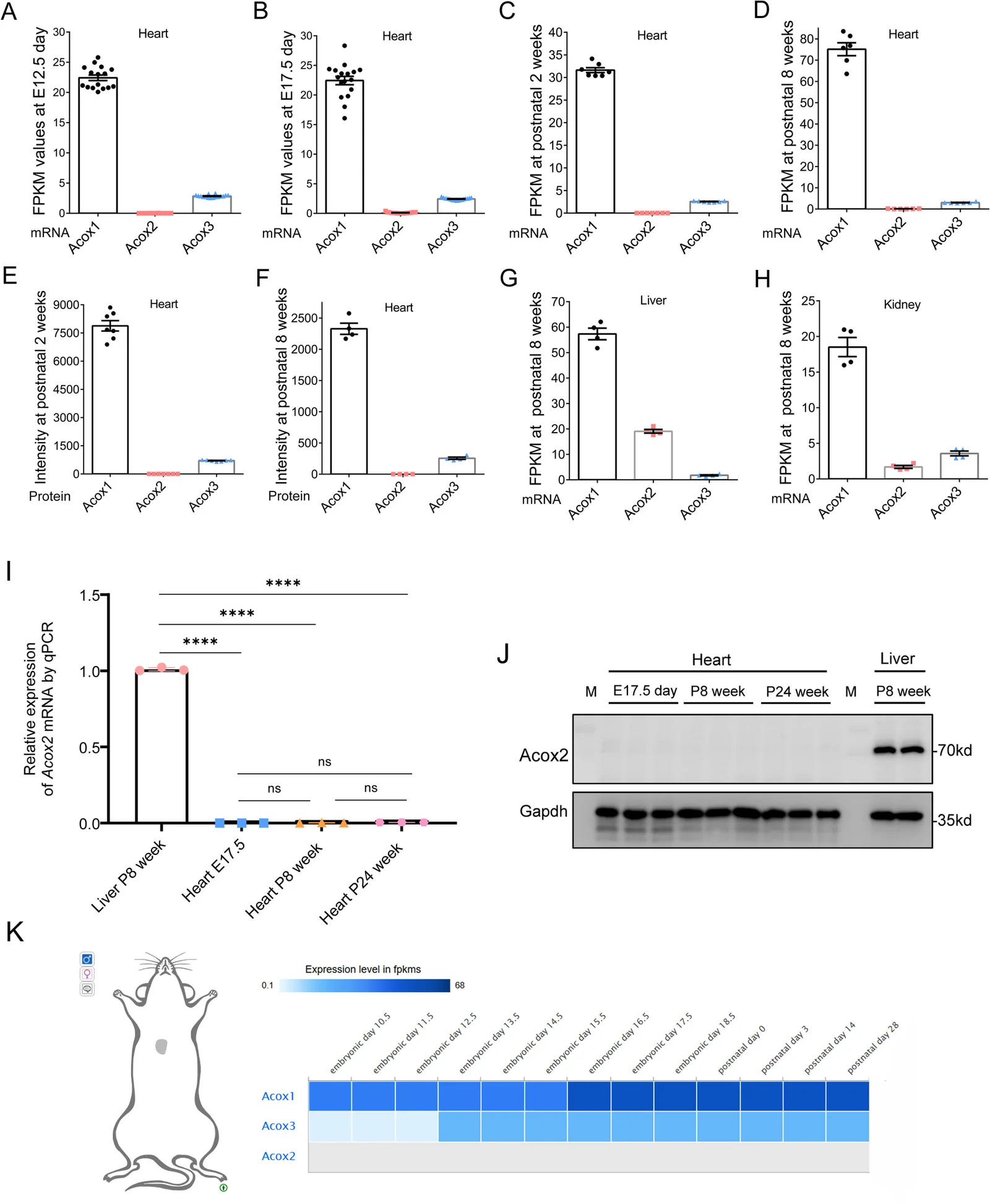
Absence of Acox2 expression in mouse heart. (**A**-**D**) FPKM (Fragments Per Kilobase of exon model per Million mapped fragments) values of three Acox family members (Acox1/2/3) in mice heart from embryonic 12.5 (*n* = 16) and 17.5 (*n* = 17) day to postnatal (P) 2 weeks (*n* = 7) and 8 weeks (*n* = 6) by RNA-seq analysis. (**E** and **F**) Normalized protein intensity of Acox1/2/3 in heart of adult mouse heart at postnatal 2 weeks (*n* = 7) and 8 weeks (*n* = 4) from TMT (Tandem mass tag) quantitative proteomic analysis. (**G** and **H**) FPKM values of three Acox family members (Acox1/2/3) in mice liver (*n* = 4) and kidney (*n* = 4) at postnatal 8 weeks by RNA-seq analysis. (**I**) Relative Acox2 level in mice heart and liver was evaluated by qPCR analysis. The data are presented as the mean ± SEM; *n* = 3, one-way ANOVA test, ****p < 0.0001. (**J**) Acox2 expression in mice heart was validated by WB analysis. Gapdh served as a loading control. M, marker for WB. A representative image from three independent experiments was displayed. (**K**) According to Mouse Genome Informatics (MGI) database records (E-MTAB-6798), RNA expression of Acox2, indicated by TPM values by RNA-seq, was below cutoff in mice heart from embryonic to adult period when compared with Acox1 and Acox3

To further elucidate the tissue and spatiotemporal specificity of Acox2 expression, we utilized multi-omics approaches, including RNA-seq and quantitative proteomics, to analyze expression patterns from the embryonic period through adulthood. FPKM (Fragments Per Kilobase of transcript per Million mapped reads) from RNA-seq and normalized protein intensity values from mass spectrometry (MS) were employed to represent the relative mRNA and protein levels of ACOX2, respectively. The results demonstrated that Acox2 mRNA levels were negligible or absent at embryonic days 12.5 (Fig. 1A) and 17.5 (Fig. 1B), as well as at postnatal weeks 2 (Fig. 1C) and 8 (Fig. 1D). The absence of the ACOX2 protein in the mouse heart was further confirmed by MS analysis at postnatal weeks 2 (Fig. 1E) and 8 (Fig. 1F). Conversely, RNA-seq data indicated that Acox2 expression is highly enriched in the liver (Fig. 1G) and moderately present in the kidney (Fig. 1H) at postnatal week 8. We further validated the lack of Acox2 expression in the murine heart from the embryonic to adult stages using qPCR (Fig. 1I) and Western blotting analysis (Fig. 1J). Furthermore, according to the Expression Atlas database, Acox2 expression is consistently reported as below the detection threshold in the mouse heart throughout its lifespan by RNA-seq analysis (Fig. 1K and Supplementary Fig.S1). Collectively, these findings confirm that ACOX2 is not expressed in the mouse heart throughout the entire lifespan.

### *ACOX2* KO mice exhibit hepatic pathology but not effect its cardiac pattern

**Fig. 2:**
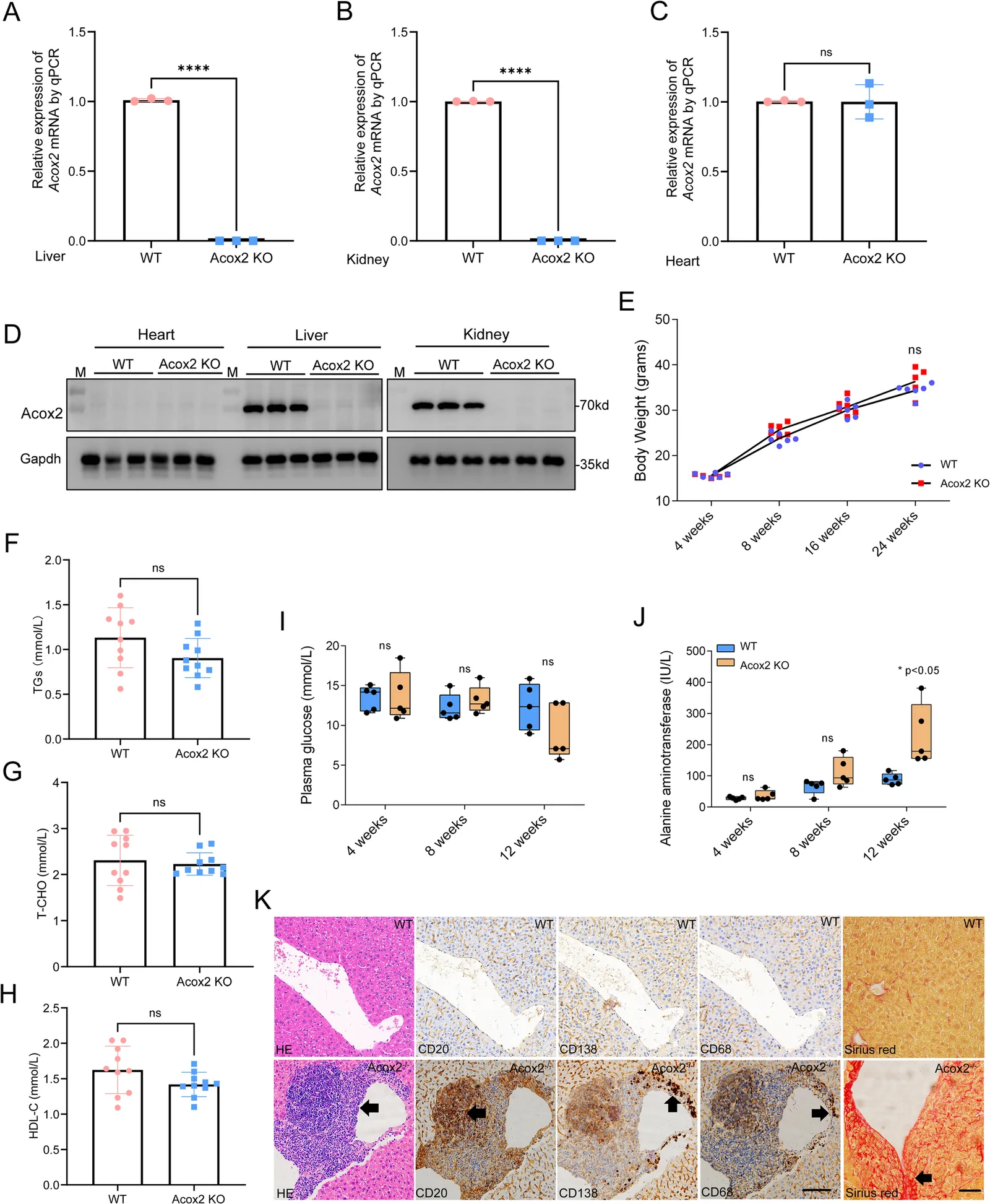
Acox2 KO induces liver inflammation and fibrosis but has no direct impact in heart. (**A**-**C**) Depletion of Acox2 expresion in liver and kidney of Acox2 KO mice was evaluated by qPCR analysis. Based on extremely low reading value (near undetectable) of Acox2 in heart, no significantly changes on mRNA level were identified between WT and Acox2 KO mice. The data are presented as the mean ± SEM; *n* = 3, one-way ANOVA test, ****p < 0.0001. (**D**) Depletion of Acox2 protein level in liver and kidney was validated by WB analysis. No band was identified for Acox2 in both WT and Acox2 KO mice heart. Gapdh served as a loading control. A representative image from three independent experiments was displayed. (**E**) Body weights of Acox2 KO and WT mice from four to 24 weeks of age (*n* = 6 per group). The data are presented as the mean ± SEM; two-tailed Student’s t-test, ns, not significant. (**F**-**H**) Three plasma lipid parameters triglycerides (TG), total cholesterol (T-CHO), and high-density lipoprotein (HDL-C) were not significantly changed in Acox2 KO mice when compared with WT controls (*n* = 10 per group). The data are presented as the mean ± SEM; two-tailed Student’s t-test, ns, not significant. (**I**-**J**) Plasma glucose and alanine aminotransferase (ALT) levels in Acox2 KO mice from four to 12 weeks of age (*n* = 5 per group). two-tailed Student’s t-test, *, *p* < 0.05; ns, not significant. (**K**) Representative images of staining for hematoxylin-eosin (HE), Sirius red and immunostaining against CD20, CD68, and CD138 on liver paraffin sections of Acox2 KO and WT mice. Black arrows indicate inflammatory lesions (H&E), positive cells (immunostaining), and hepatic fibrosis (Sirius red staining). Representative images were displayed as indicated. Scale bar, 100 μm

We previously generated Acox2 knockout (KO) mice, which recapitulate the genetic consequences of the homozygous stop-gain mutation (p.Y69X) identified in patients [21]. We performed qPCR and Western blot analyses to confirm the depletion of ACOX2 expression in the liver (Fig. 2A) and kidney (Fig. 2B) of Acox2 KO mice compared with wild-type (WT) controls. Due to extremely low, nearly undetectable reading signals in the heart of both Acox2 KO and WT mice by qPCR, no significant changes in mRNA levels were observed (Fig. 2C). Consistently, as ACOX2 is naturally absent in the heart, the protein remained undetectable in both genotypes (Fig. 2D). Given the tissue-specific expression pattern of ACOX2, we evaluated potential abnormalities in the liver and kidney. We observed no significant differences in body weight (Fig. 2E) and plasma lipid parameters including triglycerides (TG) (Fig. 2F), total cholesterol (T-CHO) (Fig. 2G), high-density lipoprotein (HDL-C) (Fig. 2H), as well as plasma glucose (Fig. 2I) in Acox2 KO mice. However, Acox2 KO mice exhibited significantly elevated serum alanine aminotransferase (ALT) levels (Fig. 2J). Consistent with previous reports [23], we observed marked inflammatory infiltration and fibrosis in the liver tissues of 4-month-old Acox2 KO mice under normal dietary conditions (Fig. 2K). Immunohistochemical staining for immune cell markers revealed that the predominant infiltrating cell type was CD20 + B-lymphocytes, followed by CD138 + and CD68 + cells (Fig. 2K). In contrast, we identified no obvious histological abnormalities in the renal cortex or medulla of Acox2 KO mice via H&E and Masson’s trichrome staining (Fig. S2A). Furthermore, serum blood urea nitrogen (BUN) (Fig. S2B) and creatinine (CREA) (Fig. S2C) levels, two primary indicators of renal function, remained within the normal range compared with WT controls at 4 months of age.

### Acox2-KO mice developed typical DCM symptoms

**Fig. 3:**
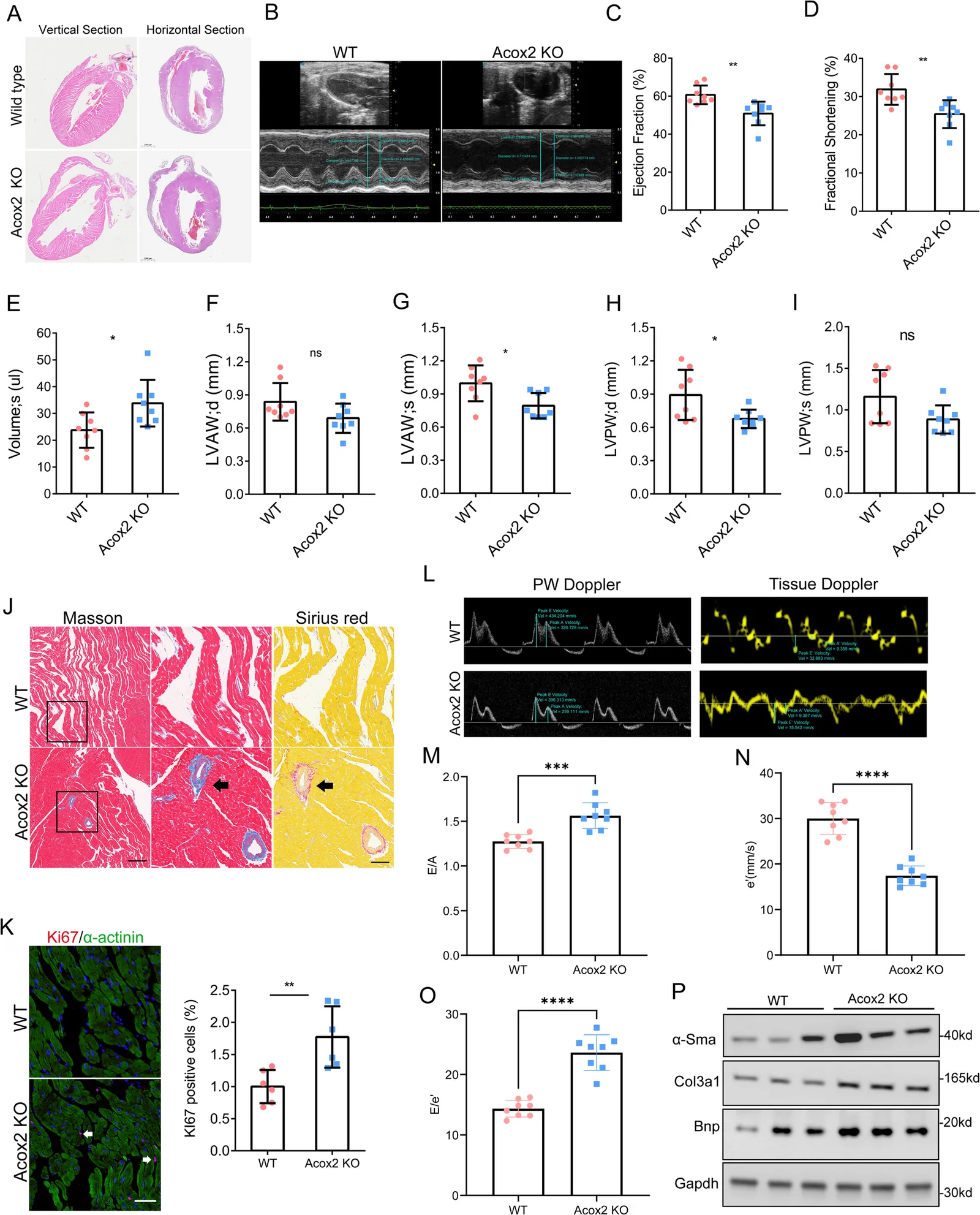
Acox2 KO mice developed phenotypes of DCM. (**A**) Overview of hematoxylin-eosin stained sections (vertical and horizontal) of Acox2 KO and WT mouse hearts. (**B**) Representative images of echocardiography in Acox2 KO mice and their WT littermates at 6 months of age. (**C**-**I**) Core parameters of echocardiographic analysis including ejection fraction (**C**), fractional shortening (**D**), volumes (**E**), diastolic (**F**) and systolic (**G**) LVAW, diastolic (**H**) and systolic (**I**) LVPW. ns, not significant, *p-value < 0.05, **p-value < 0.01 (two-tailed Student’s t-test; *n* = 8). d, diastolic; s, systolic. (**J**) Representative images of Masson’s trichrome and Sirius red staining of paraffin-embedded sections of mouse heart tissues. Fibrotic areas are boxed to show the enlarged region in Acox2 KO mice. Scale bar, 200 μm (left) and 50 μm (right). (**K**) Representative images of Ki67 staining in Acox2 KO and WT mice hearts. Cardiomyocyte specificity was confirmed by co-staining with α-actinin. Scale bar, 50 μm. Ki67-positive cardiomyocytes (white arrows) are quantified and presented as the mean ± SEM; two-tailed Student’s t test, *n* = 6. **p < 0.01. (**L**) Representative images of Pulsed-Wave (PW) Doppler of mitral valve inflow (left panels) and Tissue Doppler Imaging of the mitral annulus (right panels) in Acox2 KO and WT mice. (**M**-**O**) Quantitative analysis of the Doppler echocardiograms. (**M**) The ratio of early (E) to late (A) ventricular filling velocities (E/A ratio). (**N**) Early diastolic mitral annular velocity (e’). (**O**) The ratio of mitral peak velocity of early filling to early diastolic mitral annular velocity (E/e’), serving as a robust surrogate marker for left ventricular filling pressure. Data are presented as mean ± SEM. two-tailed Student’s t test, *n* = 8. ***p < 0.001;****p < 0.0001. (**P**) WB analysis of biomarkers for DCM and fibrosis, including Col3a1, Bnp, and Sma in WT and Acox2 KO mice. A representative image from three independent experiments was displayed

The fact that Acox2 has no protein expression in mice heart initially guides us to hypothesize that Acox2 KO would not affect cardiac function. Compared with WT controls, the echocardiogram parameters of Acox2 KO mice is not significantly changed at the age of 2 months. However, at the age of 6 months, we observed a larger heart volume through anatomical sampling and hematoxylin-eosin staining (Fig. 3A), and echocardiography analysis (Fig. 3B) revealed decreased left ventricular (LV) fractional shortening, ejection fractions, and LV anterior-wall thicknesses in Acox2 KO mice when compared to those in WT littermates (n = 8) (Fig. 3C – I). Masson’s trichrome and Sirius Red staining provided evidence of myocardial fibrosis in Acox2 KO mice (Fig. 3J). Ki67 staining indicated relative enhanced cardiomyocyte proliferation (Fig. 3K), and TUNEL staining showed no significant changes for myocardial DNA damage in Acox2 KO mice (Fig.S2D). We also conducted comprehensive Pulsed-Wave (PW) Doppler and Tissue Doppler Imaging (TDI) on the mice to assess hemodynamics and atrioventricular (AV/mitral) valve annulus velocities (Fig. 3L). Specifically, mitral inflow velocities (E and A waves) and mitral annular movement velocity (e’) was calculated. Compared with WT controls, the Acox2 KO mice exhibit a significantly reduced e’ velocity, alongside a markedly elevated E/A ratio and E/e’ ratio compared to WT mice (Fig. 3M-O). Western blot analysis further demonstrated that classical biomarkers of HF and fibrosis, including α-SMA, Bnp and Col3a1 were upregulated in the Acox2 KO mice (Fig. 3P). These findings strongly indicated that cardiac function was substantially compromised in the Acox2 mice. However, since the classical plasma lipid parameters were not significantly changed, Acox2 KO mice might not exactly resembles the cardiac dysfunction as high-fat diet induced mice model.

### Acox2 KO has limited effect on cardiac proteome in mice

**Fig. 4:**
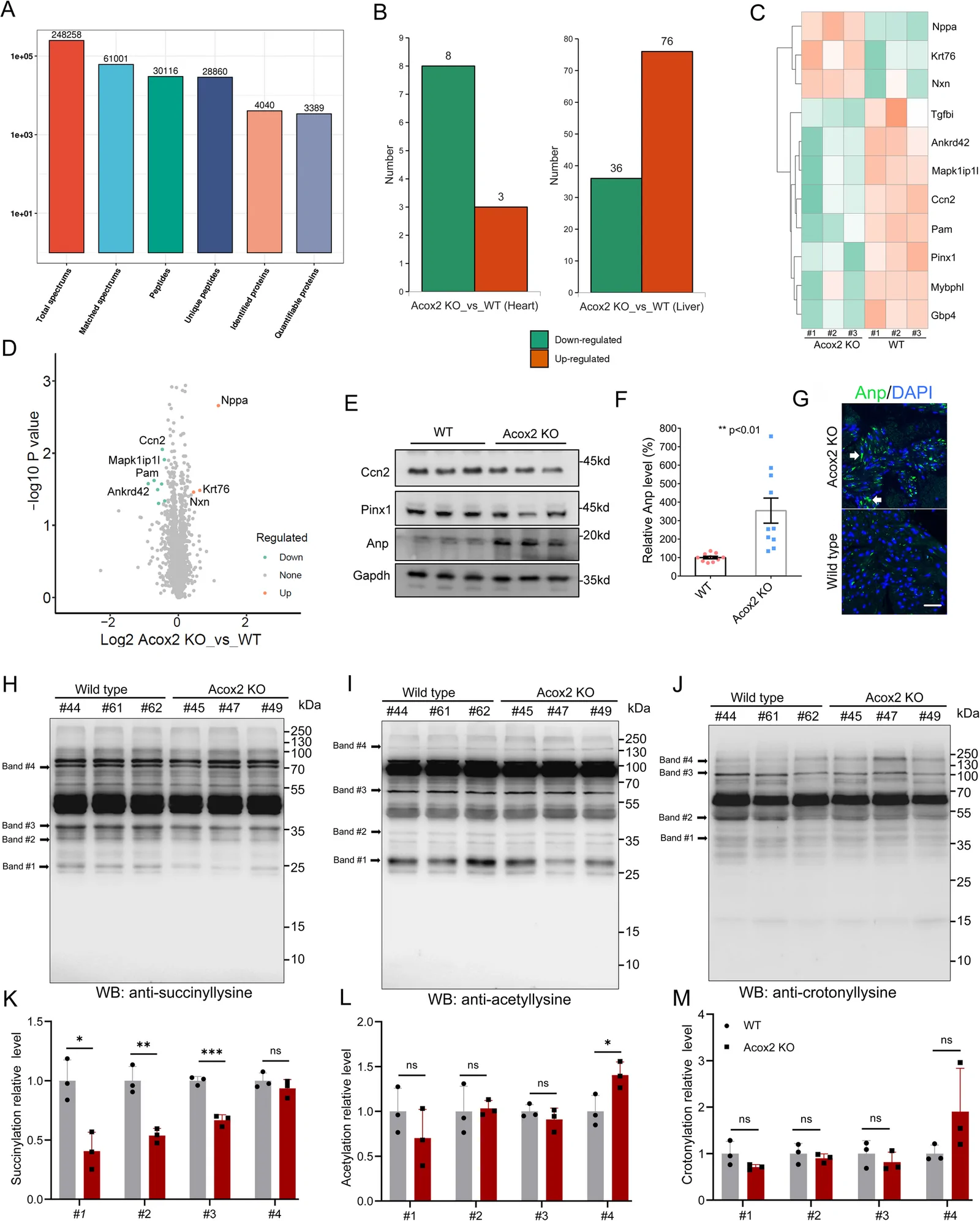
Acox2 KO leads to decreased Suc levels in mice heart but displayed limited effect on cardiac proteome. (**A**) Summary of TMT intensity of global quantitative proteomic analysis of 6 heart tissues of WT and Acox2 KO mice (*n* = 3 per group). (**B**) The number of differentially expressed genes(DEPs) (fold-change > 1.3) in two comparison groups, Acox2 KO vs. WT in heart, and Acox2 vs. WT in liver. (**C**) Heatmap of 11 DEPs in Acox2 KO mice heart (fold-change > 1.3, *p* < 0.05). (**D**) Volcano plots showing eight downregulated and three upregulated in Acox2 KO mice heart. Up- and down-regulated proteins are highlighted in red and green, respectively. (**E**) The expression of Ccn2, Pinx1, and Nppa (Anp) was then validated by WB analysis in Acox2 KO mice heart. (**F**) Quantification data of Anp expression level, a classical HF biomarker, when normalized against Gapdh. Data are presented as mean ± SEM, two-tailed Student’s t test; *n* = 10, **p value < 0.01. (**G**) Immunofluorescence staining to indicate elevated ANP (while arrows) in Acox2 KO mice heart. Scale bar, 50 μm. (**H**-**J**) Overview of anti-succinyl lysine (H),-acetyl lysine (**I**), and -crotonyl lysine (**J**) in heart tissues of Acox2 KO and WT (*n* = 3) mice using corresponding pan-antibodies by western blots. Representative images from three independent experiments was displayed. (**K**-**M**) Quantification of the western blot bands in (**H**-**J**), respectively. For each protein modification, four viable bands were choosen for quantification as indicated. Data are presented as the mean ± SEM; two-tailed Student’s t test, *n* = 3. *p < 0.05, **p < 0.01

To investigate the mechanism by which Acox2 KO contributes to cardiac phenotypes, we performed global tandem mass tagging (TMT) quantitative proteomic analysis on heart tissues of Acox2 KO mice and their WT littermates (*n* = 3) at 6 months of age (Fig. 4A). This analysis revealed 30,116 peptides mapping to 4,040 proteins. Principal-component analysis was unable to separated each of the 6 heart samples into two categories. We then profile the significant differentially expressed proteins (DEPs) between WT and Acox2 KO mice (fold-change > 1.3, *p* < 0.05). As expected, we did not identify signals on Acox2 by MS analysis and a total of 11 DEPs (8 downregulated and 3 upregulated) were identified in Acox2 KO mice hearts (Fig. 4B-D). We then compare the effect of Acox2 KO on hepatic and cardiac proteome via profiling TMT quantitative proteomics data. In the liver tissue, a total of 112 DEPs, 76 upregulated and 36 downregulated (fold-change > 1.3, *p* < 0.05), were identified in Acox2 KO mice when compared with WT controls at 4 months of age (Fig. 4B and supplementary file). In contrast to the hepatic proteome, these results suggest that Acox2 KO has a limited, or at least relatively minor, effect on the cardiac proteome in mice. We also validate the expression of two proteins Ccn2 (fold-change = 1.38) and Pinx1 (fold-change = 1.49), and the reduction of these two proteins in Acox2 KO mice heart is moderate (Fig. 4E). Notably, Anp protein encoded by *NPPA*, another well-known marker for HF, was significantly increased in Acox2-KO hearts, as demonstrated by western blotting (Fig. 4E-F) and immunofluorescence (Fig. 4G) analyses.

### Decreased Suc levels in Acox2 KO mice

Given the minimal effect of *Acox2* KO on cardiac proteome, we postulate that PTMs mediated metabolic reprogramming might play a role in the cardiac phenotypes observed in Acox2-KO mice. Antibody screening for various PTMs, including succinylation (Suc) (Fig. 4H and S3A), acetylation (Fig. 4I and S3B), crotonylation (Fig. 4J and S3C) and ubiquitination (Fig.S3D) with relative long and short exposure time. Full length coomassie blue staining for each blot served as loading control to verify equal loading (Fig.S3). We then quantified the viable bands with higher intensity cross samples to calculate the potential changes on different PTMs (Fig. 4K-M and S3E). Notably, We identified three significantly decreased WB bands for Suc signals that located at near 20 and 35 kDa, respectively, and one slightly upregulated WB band for acetylation signals at approxiamate 130 kDa in Acox2 KO hearts compared to WT hearts at the age of 6 months. No significant changes were detected for crotonylation and ubiquitination levels in Acox2 KO mice. These findings indicated that Suc levels on non-histone proteins were deceased in hearts of Acox2 KO mice.

### Decreased Suc sites were enriched on myofibril and mitochondrial proteins in Acox2 KO mice

**Fig. 5:**
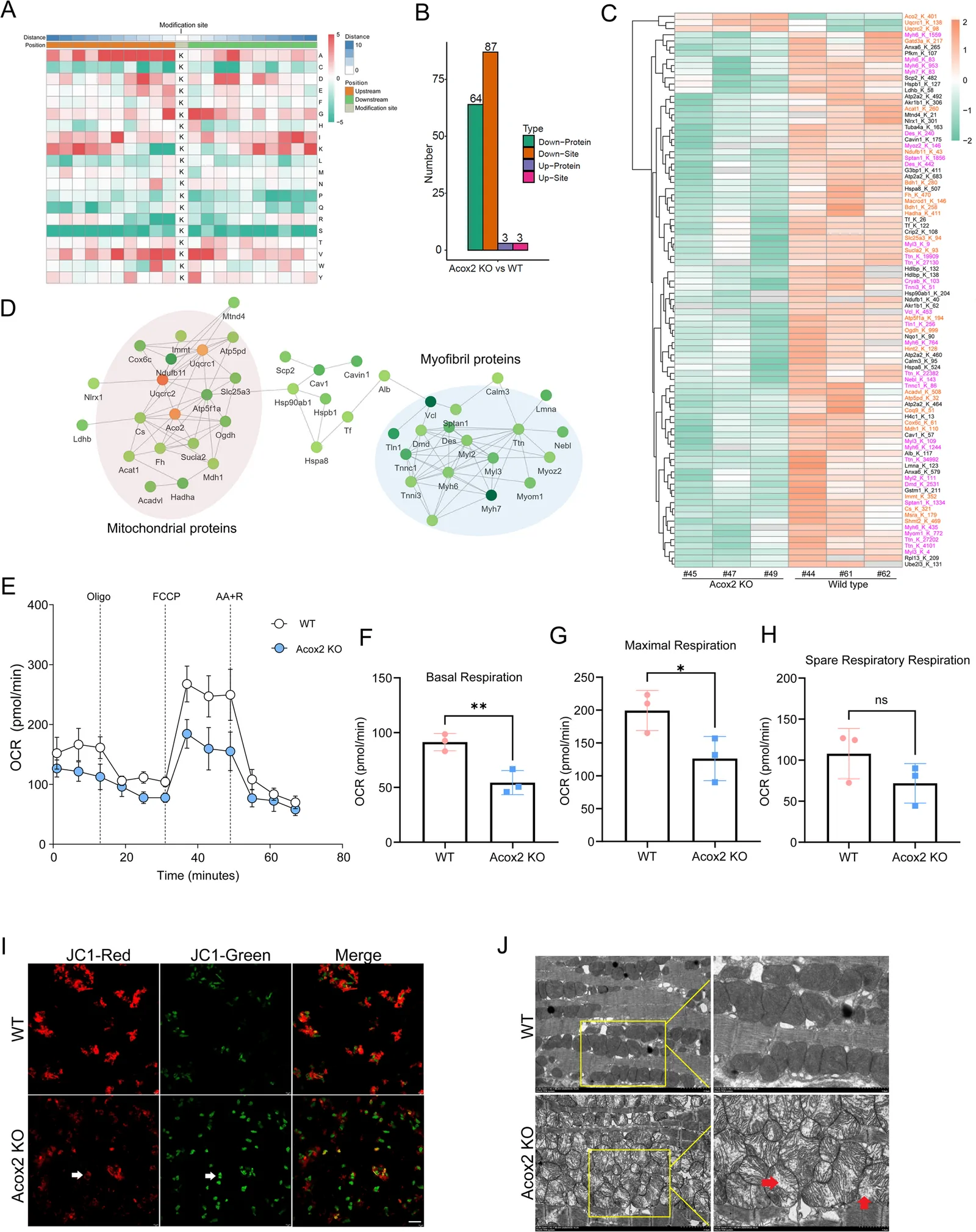
Decreased Suc sites were enriched on mitochondrial proteins accompanied by impaired mitochondrial function in Acox2-KO mice. (**A**) Motif analysis of all identified succinylated sites. (**B**) 87 down-regulates and 3 up-regulated sites were identified in Acox2 KO mice heart (fold-change > 1.3, *p* < 0.05). (**C**) Heatmap showing profiles of all 90 significantly changed Suc sites after normalization according to quantitative proteomics analysis. The sites on mitochondria and myofibril are highlighted in orange and purple, respectively. (**D**) Top 50 changed succinylation proteins were searched against the STRING database for protein-protein interactions (PPI). Interaction network form STRING was then visualised in R package networkD3. (**E**) Seahorse analysis of mitochondrial respiration in primary CMs from WT and Acox2 KO mice. Representative oxygen consumption rate (OCR) traces and quantification of basal respiration, spare respiratory capacity, and maximal respiration are shown. Data are presented as mean ± SEM, two-tailed Student’s t test, *n* = 3, *p < 0.05, **p < 0.01. (**I**) Representative images of JC-1 dye staining in primary CMs from Acox2 KO and WT mice by immunofluorescence assay. Scale bar, 100 μm. (**J**) Representative images of scanning electron microscopy (SEM) analysis indicated morphological abnormalities of mitochondria in the hearts of Acox2 KO mice. Mitochondrial swelling and vacuolization are indicated by red arrows in Acox2 KO mice. Scale bar, 2.0 and 1.0 μm

To precisely determine Suc changes in Acox2 KO mouse hearts developed HF, a global succinylome analysis was performed using heart tissues collected from Acox2 KO mice (*n* = 3) and WT littermates (*n* = 3) when they were 6 months old. A total of 3,451 quantifiable Suc sites on 688 proteins were identified and normalized to quantitative proteomic data for each sample. We analyzed the flanking sequences of these sites and found that glycine (G) and valine (V) were overrepresented, whereas serine (S) and cysteine (C) were present at lower levels than expected at the − 1 and + 1 positions surrounding the K-Suc sites (Fig. 5A). We identified 87 significantly down-regulated Suc sites (fold-change > 1.3; *p* < 0.05) in 64 proteins in Acox2-KO mice, while only three Suc sites on mitochondrial proteins (Aco2, Uqcrc1 and Uqcrc2) were moderately up-regulated (1.3 < fold-change < 1.5) when compared to the corresponding Suc levels in the WT group (Fig. 5B and C). Notably, over 60% of decreased Suc sites were located on myofibril and mitochondrial proteins (Fig. 5D). 22 down-regulated Suc sites were identified in 21 mitochondrial proteins, including Suc sites in Acat1 (K260), Atp5f1a (K194), Bdh1 (K258 and K280), Cs (K321), Fh (K470), Hadha (K411), Hint2 (K128), Ndufb11 (K43), Ogdh (K999), and Sucla2 (K94), and 30 down-regulated Suc sites on 16 proteins were located in myofibril proteins closely associated with cardiomyopathy, including Tnnc1 (K86), Tnni3 (K51), Vcl (K453), Myh6 (K83, K435, K764, K953, K1244, and K1559), Myh7 (K83), Myl2 (K111), and Myl3 (K4, K9, and K109) (Supplementary Table S1).

Given that a significant number of mitochondrial proteins display decreased Suc sites, we next assess mitochondrial function in heart tissue of Acox2 KO mice. The Seahorse XFe96 Analyzer was used to measure mitochondrial oxygen consumption rate (OCR) by following the standard procedure (Fig. 5E). Primary cardiomyocytes (CMs) were extracted from the hearts of Acox2 KO and WT mice at the age of 16-week. Primary CMs from the hearts of Acox2 KO mice exhibited significantly reduced basal and maximal respiration, with a mild decrease in spare respiratory capacity (Fig. 5F-H). We next perform the immunofluorescence assay with JC-1 dye to evaluate the mitochondrial membrane potential in primary CMs. We observed a significant decrease in the red-to-green fluorescence intensity ratio in CMs of Acox2 KO mice compared to WT controls (Fig. 5I). Subsequent scanning electron microscopy (SEM) analysis indicated mitochondria in the hearts of Acox2 KO mice exhibited morphological abnormalities including edema, evident damage, increased mitochondrial number and aggregation, swelling and enlargement with irregular shapes (Fig. 5J). These finding indicated abnormal mitochondrial function accompanied by impaired ATP synthesis in heart of Acox2 KO mice.

**Fig. 6:**
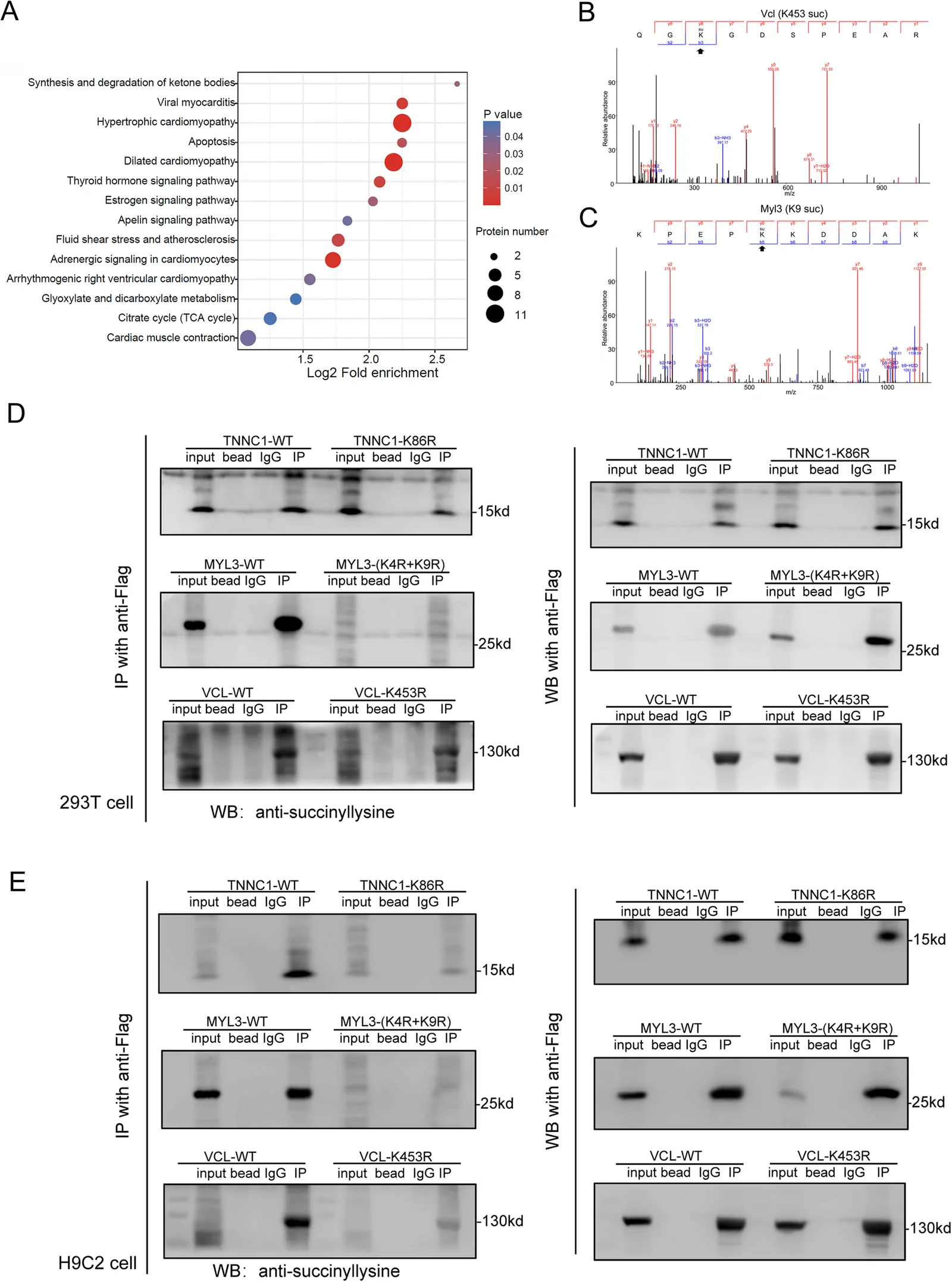
Decreased Suc sites on myofibril proteins were potentially correlated with DCM and HCM in Acox2 KO mice. (**A**) Enrichment analysis of KEGG pathways for down-regulated succinylation proteins in Acox2 KO mice (Fisher’s exact test; *p* < 0.05). (B-C) The MS/MS spectrum of the K453 Suc modified site in Vcl (**B**) and the K9 Suc modified site in Myl3 (**C**). (**D**-**E**) Replacing lysine with arginine at Suc sites on VCL (K453R), TNNC1 (K86R), and MYL3 (K4R+K9R) led to decreased Suc levels in 293T (**D**) and H9C2 (**E**) cells. Cells were transfected with Flag-tagged WT and point mutants recombinant proteins, and cell lysates were then immunoprecipitated with an anti-Flag antibody, followed by immunoblotting with a pan-Suc antibody as indicated. Bead-only and isotype (IgG) served as control group. For each western blot, representative images from three independent experiments were displayed

Since many decreased Suc sites were identified on cardiomyopathy related proteins including Myl3, Myh6, Myh7, Vcl, and Tnnc1, KEGG pathway terms “hypertrophic cardiomyopathy” and “dilated cardiomyopathy” were the most significantly enriched terms identified when analyzing these proteins (Fig. 6A-C and S4). We next generated Myl3, Vcl and Tnnc1 mutants in which lysine Suc sites at positions 4 and 9 of Myl3, position 453 of Vcl, and position 86 of Tnnc1, were simultaneously mutated to arginine by site-specific mutagenesis to mimic the desuccinylated status. The Suc signals on Myl3, Vcl and Tnnc1 were validated by immunoprecipitation assays in HEK-293T and H9C2 cell lines overexpressing Flag-tagged recombinant proteins (Fig. 6D-E and S5). Of note, mutating lysine to arginine in Suc sites of Myl3 (K4R and K9R), Vcl (K453R) or Tnnc1 (K86R) reduces Suc levels to various degrees in vitro (Fig. 6D - E), which further validate the data reliability of succinylome analysis.

### Accumulated serum C27 cholesterol derivatives and decreased cardiac succinyl-CoA in Acox2 KO mice

**Fig. 7:**
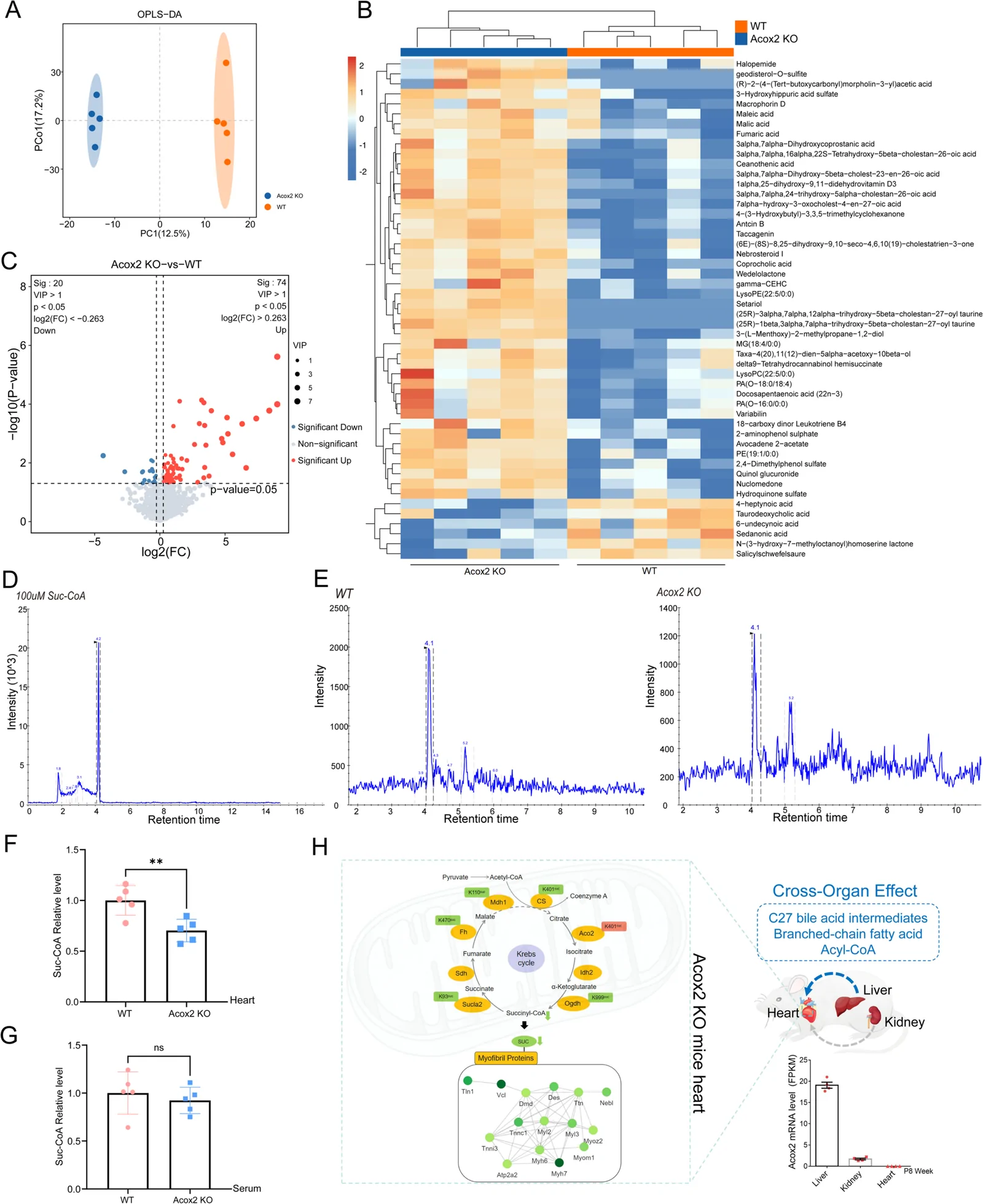
Accumulated serum C27 bile acid intermediates and decreased cardiac succinyl-CoA in Acox2 KO mice. (**A**) OPLS-DA analysis effectively separated 10 plasma into 2 distinct categories (*n* = 5 per group). (**B**) Heatmap of Top 50 significantly different metabolites (SDMs) (VIP > 1; *p* < 0.05; FC > 1.2) in the serum samples of Acox2 KO mice when compared with WT mice. (**C**) Volcano plots showing the SDMs in the Acox2 KO vs. WT comparison group. Up- and downregulated proteins are highlighted in red and blue, respectively. The Top10 upregulated SDMs were mostly referring to C27 and C29 bile acids intermediates. (**D**) Representative mass spectrometry peaks of Succinyl-CoA *(Suc-coA)* standards at the concentration of 100 μm. (**E**) Representative mass spectrometry peaks showing Succinyl-CoA intensities in heart samples from Acox2 KO and WT mice. (**F**-**G**) Quantification analysis of relative Succinyl-CoA levels in heart and serum samples of Acox2 KO and WT mice. Data are presented as mean ± SEM (*n* = 5 mice). Statistical significance was determined using a two-tailed Student’s t-test: **p < 0.01. (**H**) Schematic representation of the proposed cross-organ metabolic mechanism of cardiac dysfunction in Acox2 KO mice. Suc, succinylation

Since absent expression of Acox2 in mice, we postpulate cardiac dysfunction in Acox2 KO mice probably induced by serum metabolites mediated that cross-organ effect. To comprehensively characterize the metabolic features in serum of Acox2 KO mice (*n* = 5) and WT control (*n* = 5), we performed global metabolomics analysis and a total of 2369 metabolites were profiled. Orthogonal partial least squares discrimination analysis (OPLS-DA) effectively separated 10 heart samples into 2 distinct categories (Fig. 7A). We identified 74 up- and 20 down-regulated significantly different metabolites (SDMs) (VIP > 1; *p* < 0.05; Fold-change > 1.2) in the serum samples of Acox2 KO mice when compared with WT Ctrls (Fig. 7B). The Top10 upregulated SDMs in volcano plot were mostly referring to C27 and C29 bile acids intermediates, including (25R)-3alpha,7alpha,12alpha-trihydroxy-5beta-cholestan-27-oyl taurine and 3alpha,7alpha,24-trihydroxy-5alpha-cholestan-26-oic acid, in Acox2 KO mice (Fig. 7C and Supplementary file). We observed the dramatically accumulation of C27 bile acids intermediates accompanied by downregulated taurodeoxycholic acids Acox2 KO mice, which faithfully recapitulate the serum biochemical changes in the patients carrying homozygous ACOX2 stop-gain mutation. We also observed dysregulated niacinamide level in the serum of Acox2 KO mice. Subsequent KEGG enrichment analysis indicated that “Citrate cycle (TCA cycle)”, “Primary bile acid biosynthesis” and “Nicotinate and nicotinamide metabolism” were enriched terms by these SDMs in serum of Acox2 KO mice. These findings suggested numerous metabolites related to bile acid metabolism and TCA cycle potentially play cross-talk regulation to effect cardiac function.

Since protein expression of the succinyltransferase CPT1A (fold-change, FC = 1.03), and de-succinylases SIRT5 (FC = 0.92) and HDAC1 (FC = 1.07) remained unchanged in Acox2 KO mouse hearts according to proteomic data, we postulated that reduced Suc levels in Acox2 KO mice were probably caused by shortage on succinyl-CoA, the primary donor for protein Suc modification. We next utilized LC-MS-based targeted analysis to measure succinyl-CoA and succinic acid levels in the serum and heart tissues of Acox2 mice (Fig. 7D and S6A-E), which revealed a significant reduction of succinyl-CoA in heart samples but not changes in serum sample (Fig. 7E-G and S6F-G). The relative intensity of succinyl-CoA from LS-MS analysis was very low in serum sample when compared with heart. These findings suggest that a scarced the succinyl donor pool occurs in pathological cardiac states in Acox2 KO mice.

## Discussion

Cross-organ effects mediated by metabolic programming are considered a significant factor contributing to secondary cardiomyopathies [27, 28]. However, the lack of effective animal models has hindered detailed mechanistic studies. Here, we have identified a naturally occurring animal model suitable for investigating inter-organ metabolic regulation, especially distinct the non-cardiac effect on cardiac phenotypes. Given the absence of Acox2 protein expression in mouse hearts from embryonic stage to adulthood, the cardiac phenotypes observed in Acox2 KO mice is a consequence of secondary cardiomyopathy resulting from a cross-organ effect, probably a type of hepato-cardial syndrome (Fig. 7H). Generally, Acox2 KO mice model provide a specialized model to indicate the predominant contribution of non-cardiac factor to cardiac phenotype. These non-cardiac factors can be classified as a combined effects derived from all other non-cardiac organs like liver and kidney, which could not be archived by conditional knock-out on single organ, which represents the main advantage of this model.

Acox2 KO mice recapitulated the hepatic phenotypes, specifically liver fibrosis, lymphocytic infiltrate and accumulated plasma C27 intermediates, seen in patients harboring ACOX2 homozygous mutations. These findings aligns with clinical observations that over 50% of patients with liver cirrhosis exhibit varying degrees of cardiomyopathic phenotypes, underscores a crucial regulatory role of liver homeostasis in adult cardiac function. Accumulated C27 bile acid intermediates was supposed to be the cause of liver damage because ursodeoxycholic acid treatment can efficiently attenuate liver damage in these patients [29]. Previous study reported cardiac dysfunction in patients with liver cirrhosis is strongly associated with increased serum bile acid concentrations by suppressing expression of Pgc1α, a key regulator of fatty acid metabolism [30]. Therefore, it seems like interrupted bile acid synthesis by Acox2 KO may be beneficial against the metabolic and pathological changes in the heart. Meanwhile, examining the direct effect of accumulated serum C27 bile acid intermediates on cardiac metabolism, especially mitochondrial function and TCA cycle, in Acox2 KO mice would be a potential avenue for mechanistic understanding of these observations.

Highly conservatism of ACOX2 across bacteria and plants suggesting that the initial biological function of Acox2 may not have been related to bile acid synthesis from an evolutionary perspective. Indeed, the results of we and other previous studies revealed potential roles of acyl-CoA oxidase family members (ACOX1/2/3) in regulation of PTMs including lysine crotonylation and acetylation [23, 31, 32]. Our recent study indicated that ACOX2 binds with MRE11 and destabilizes the MRE11-RAD50-NBS1 complex [33]. In particular, ACOX2 overexpression in adipose tissue enhanced thermogenesis independently of UCP1 and improved metabolic homeostasis [34]. All these findings demonstrate Acox2 plays crucial role through a bile acid independent manner on metabolic regulation.

The main limitation of our study is lack of the precise mechanism to explain the regulatory connection between Acox2 KO and succinylation. So far, there is no direct evidence prove Acox2 could directly participate in Suc-CoA metabolism which is a critical step of TCA cycle in mitochondrion. We concluded that the downregulated succinylation in Acox2 KO mice is due to levels decreased of the Succinyl donor pool since protein level of succinyltransferases and eraser were not significantly altered. Previous studies have established that succinylation intensity on non-histone proteins play key role in the progress of HF in patients and mice models [15, 16]. Activity changes on mitochondrial proteins could directly effect the succinylation levels in TCA cycle. IDH mutations promote hypersuccinylation that impairs mitochondria respiration [35]. We identified decreased Suc sites on 6 mitochondrial protein (Sucla2_K93, Cs_K321, Aco2_K401, Ogdh_K999, Fh_K470 and Mdh1_K110) that participate in Citrate cycle and succinyl-CoA production. Sucla2 is an ATP-specific Succinyl-CoA synthetase (SCS) beta subunit that dimerizes with the alpha subunit to form SCS complex that hydrolyzes ATP to reversible convert succinate to succinyl-CoA. SUCLA2 mutations lead to global protein succinylation contributing to the pathomechanism of a hereditary mitochondrial disease [36], suggesting the activity of SUCLA2 was directly involved in succinylation regulation. The protein level of Sucla2 was significantly decreased in myocardial infarction mice [15]. Moreover, SIRT5 mediated succinylation of SUCLA2 regulates TCA cycle dysfunction and mitochondrial damage in pancreatic acinar cells [37]. Ogdh constitute one subunit of the 2-oxoglutarate dehydrogenase complex that catalyzes the overall conversion of 2-oxoglutarate (alpha-ketoglutarate) to succinyl-CoA during the Krebs cycle. Recent study found that desuccinylation of OGDH by SIRT5 inhibits the activity of OGDH complex [38]. However, all these metabolic and succinylation changes might be just secondary to mitochondrial abnormality and cardiac dysfunction in Acox2 KO mice, not a primary drive factor.

The relative concentration of Suc-coA in heart is magnitude higher than it in plasma, therefore, it is impossible the plasma Suc-coA could directly affect its cardiac concentration. Based on phenotypes on liver and kidney in Acox2 KO mice, we assume abnormal metabolites from hepatic dysfunction provide the primary drive factor that circulating to heart tissue to take a role. Therefore, a reasonable explanation was plasma circulation accumulated C27 intermediates cause mitochondrial damage in cardiomyocytes in Acox2 KO mice, decreased succinylation then further aggravate the pathological process of heart failure.

## Conclusions

Here our findings underscore the strong correlation between decreased Suc levels and impaired cardiac function. Given the consistently lower levels of succinylation on non-histone proteins, including myofibrillar and mitochondrial proteins, observed in multiple mouse models of cardiomyopathy and heart failure, enhancing serum succinyl-CoA levels represents a potential therapeutic strategy to improve cardiac function. Overall, our results suggested that the development of secondary cardiomyopathy in Acox2 KO mice, likely mediated through a cross-organ metabolic effect, underscores the critical role of bile acids in the pathogenesis of hepato-cardiac disorders such as cirrhotic cardiomyopathy, and highlights the need for considering systemic metabolic interactions between organs in cardiac disease.

## Supplementary Information

Below is the link to the electronic supplementary material.

Supplementary Material 1

Supplementary Material 2

Supplementary Material 3

Supplementary Material 4

## Data Availability

The raw RNA sequencing data reported in this study have been deposited at the Genome Sequence Archive in National Genomics Data Center, China National Center for Bioinformation (GSA: PRJCA067074) that are publicly accessible at https://ngdc.cncb.ac.cn. Other data supporting the findings of this study are available from the corresponding authors upon reasonable request.

## Abbreviations

Acox2: Acyl-CoA oxidase 2
ANP: Atrial natriuretic peptide
Cs: Citrate synthase
Ctrl: Control
DCM: Dilated cardiomyopathy
DEPs: Differentially expressed proteins
Fh: Fumarate hydratase
GO: Gene Ontology
HF: Heart failure
KEGG: Kyoto Encyclopedia of Genes and Genomes
KO: Knockout
LV: Left ventricle
MS: Mass spectrometer
Mdh1: Malate dehydrogenase 1
Myh6: Myosin heavy chain 6
Myh7: Myosin heavy chain 7
Myl2: Myosin light chain 2
Myl3: Myosin light chain 3
PTM: Post-translational modification
SDMs: Significantly different metabolites
Suc: Succinylation
Sucla2: Succinate-CoA ligase ADP-forming subunit beta
Ogdh: Oxoglutarate dehydrogenase
Tnnc1: Troponin C1, slow skeletal and cardiac type
Tnni3: Troponin I3, cardiac type
Vcl: Vinculin

## References

[1] Schultheiss HP, Fairweather D, Caforio ALP, Escher F, Hershberger RE, Lipshultz SE, Liu PP, Matsumori A, Mazzanti A, McMurray J, Priori SG. Dilated cardiomyopathy. Nat Rev Dis Primers. 2019;5(1):32. 10.1038/s41572-019-0084-1 PMC7096917 31073128

[2] Nieminen MS, Harjola VP, Hochadel M, Drexler H, Komajda M, Brutsaert D, Dickstein K, Ponikowski P, Tavazzi L, Follath F, Lopez-Sendon JL. Gender related differences in patients presenting with acute heart failure. Results from EuroHeart Failure Survey II. Eur J Heart Fail. 2008;10(2):140–8. 10.1016/j.ejheart.2007.12.012 18279769

[3] Lund LH, Edwards LB, Dipchand AI, Goldfarb S, Kucheryavaya AY, Levvey BJ, Meiser B, Rossano JW, Yusen RD, Stehlik J, International Society for Heart and Lung Transplantation. The Registry of the International Society for Heart and Lung Transplantation: Thirty-third Adult Heart Transplantation Report-2016; Focus Theme: Primary Diagnostic Indications for Transplant. J Heart Lung Transpl. 2016;35(10):1158–69. 10.1016/j.healun.2016.08.017 27772668

[4] Waagstein F. Beta-adrenergic blockade in dilated cardiomyopathy, ischemic cardiomyopathy, and other secondary cardiomyopathies. Heart Vessels Suppl. 1991;6:18–28. 10.1007/BF01752532 1687923

[5] Møller S, Henriksen JH. Cirrhotic cardiomyopathy. J Hepatol. 2010;53(1):179–90. 10.1016/j.jhep.2010.02.023 20462649

[6] Wiese S, Hove JD, Bendtsen F, Møller S. Cirrhotic cardiomyopathy: pathogenesis and clinical relevance. Nat Rev Gastroenterol Hepatol. 2014;11(3):177–86. 10.1038/nrgastro.2013.210 24217347

[7] Izzy M, VanWagner LB, Lin G, Altieri M, Findlay JY, Oh JK, et al. Redefining Cirrhotic Cardiomyopathy for the Modern Era. Hepatology. 2020;71(1):334–45. 10.1002/hep.30875 PMC7288530 31342529

[8] Torregrosa M, Aguadé S, Dos L, Segura R, Gónzalez A, Evangelista A, et al. Cardiac alterations in cirrhosis: reversibility after liver transplantation. J Hepatol. 2005;42(1):68–74. 10.1016/j.jhep.2004.09.008 15629509

[9] Tan M, Luo H, Lee S, Jin F, Yang JS, Montellier E, Buchou T, Cheng Z, Rousseaux S, Rajagopal N, Lu Z, Ye Z, Zhu Q, Wysocka J, Ye Y, Khochbin S, Ren B, Zhao Y. Identification of 67 histone marks and histone lysine crotonylation as a new type of histone modification. Cell. 2011;146(6):1016–28. 10.1016/j.cell.2011.08.008 PMC3176443 21925322

[10] Zhao S, Xu W, Jiang W, Yu W, Lin Y, Zhang T, Yao J, Zhou L, Zeng Y, Li H, Li Y, Shi J, An W, Hancock SM, He F, Qin L, Chin J, Yang P, Chen X, Lei Q, Xiong Y, Guan KL. Regulation of cellular metabolism by protein lysine acetylation. Science. 2010;327(5968):1000–4. 10.1126/science.1179689 PMC3232675 20167786

[11] Zhang Z, Tan M, Xie Z, Dai L, Chen Y, Zhao Y. Identification of lysine succinylation as a new post-translational modification. Nat Chem Biol. 2011;7(1):58–63. 10.1038/nchembio.495 PMC3065206 21151122

[12] Sadhukhan S, Liu X, Ryu D, Nelson OD, Stupinski JA, Li Z, Chen W, Zhang S, Weiss RS, Locasale JW, Auwerx J, Lin H. Metabolomics-assisted proteomics identifies succinylation and SIRT5 as important regulators of cardiac function. Proc Natl Acad Sci U S A. 2016;113(16):4320–5. 10.1073/pnas.1519858113 PMC4843474 27051063

[13] Yang L, Miao S, Zhang J, Wang P, Liu G, Wang J. The growing landscape of succinylation links metabolism and heart disease. Epigenomics. 2021;13(4):319–33. 10.2217/epi-2020-0273 33605156

[14] Zhang Y, Yao C, Chen Y, Cai K, Lu Z, Wu B, Yu K, Shi Y, Zhao J, Zhou X. G3BP1 succinylation at K413 is critical for cardiac function by modulating PI3K-AKT-mTOR signal axis. Adv Sci (Weinh). 2026;13(43):e19856. 10.1002/advs.202519856 PMC13335847 42107065

[15] Takada S, Maekawa S, Furihata T, Kakutani N, Setoyama D, Ueda K, et al. Succinyl-CoA-based energy metabolism dysfunction in chronic heart failure. Proc Natl Acad Sci U S A. 2022;119(41):e2203628119. 10.1073/pnas.2203628119 PMC9564216 36201541

[16] Ali HR, Michel CR, Lin YH, McKinsey TA, Jeong MY, Ambardekar AV, et al. Defining decreased protein succinylation of failing human cardiac myofibrils in ischemic cardiomyopathy. J Mol Cell Cardiol. 2020;138:304–17. 10.1016/j.yjmcc.2019.11.159 PMC7058372 31836543

[17] Vanhove GF, Van Veldhoven PP, Fransen M, Denis S, Eyssen HJ, Wanders RJ, et al. The CoA esters of 2-methyl-branched chain fatty acids and of the bile acid intermediates di- and trihydroxycoprostanic acids are oxidized by one single peroxisomal branched chain acyl-CoA oxidase in human liver and kidney. J Biol Chem. 1993;268(14):10335–44. 8387517

[18] Reddy JK, Hashimoto T. Peroxisomal beta-oxidation and peroxisome proliferator-activated receptor alpha: an adaptive metabolic system. Annu Rev Nutr. 2001;21:193–230. 10.1146/annurev.nutr.21.1.193 11375435

[19] Dave A, Hernández ML, He Z, Andriotis VM, Vaistij FE, Larson TR, Graham IA. 12-oxo-phytodienoic acid accumulation during seed development represses seed germination in Arabidopsis. Plant Cell. 2011;23(2):583–99. 10.1105/tpc.110.081489 PMC3077774 21335376

[20] Monte MJ, Alonso-Peña M, Briz O, Herraez E, Berasain C, Argemi J, Prieto J, Marin JJG. ACOX2 deficiency: An inborn error of bile acid synthesis identified in an adolescent with persistent hypertransaminasemia. J Hepatol. 2017;66(3):581–8. 10.1016/j.jhep.2016.11.005 27884763

[21] Vilarinho S, Sari S, Mazzacuva F, Bilgüvar K, Esendagli-Yilmaz G, Jain D, Akyol G, Dalgiç B, Günel M, Clayton PT, Lifton RP. ACOX2 deficiency: A disorder of bile acid synthesis with transaminase elevation, liver fibrosis, ataxia, and cognitive impairment. Proc Natl Acad Sci U S A. 2016;113(40):11289–93. 10.1073/pnas.1613228113 PMC5056113 27647924

[22] Zhang Y, Lu Z, Zeng W, Zhao J, Zhou X. Two sides of NNMT in alcoholic and non-alcoholic fatty liver development. J Hepatol. 2021;74(5):1250–3. 10.1016/j.jhep.2020.11.049 33340570

[23] Zhang Y, Chen Y, Zhang Z, Tao X, Xu S, Zhang X, Zurashvili T, Lu Z, Bayascas JR, Jin L, Zhao J, Zhou X. Acox2 is a regulator of lysine crotonylation that mediates hepatic metabolic homeostasis in mice. Cell Death Dis. 2022;13(3):279. 10.1038/s41419-022-04725-9 PMC8964741 35351852

[24] Zhou X, Wang H. ACOX2 deficiency in primary malignant cardiac tumors. Proc Natl Acad Sci U S A. 2017;114(18):E3590–1. 10.1073/pnas.1701212114 PMC5422777 28400508

[25] Zhou X, Xu M, Zeng W, Chen Z, Lu G, Gong Y, Finnell RH, Xiao H, Qiao B, Wang H. Combined effects of FH (E404D) and ACOX2 (R409H) cause metabolic defects in primary cardiac malignant tumor. Cell Death Discov. 2018;4:18. 10.1038/s41420-018-0072-3 PMC6056498 30062063

[26] Moreno P, Fexova S, George N, Manning JR, Miao Z, Mohammed S, et al. Expression Atlas update: gene and protein expression in multiple species. Nucleic Acids Res. 2022;50(D1):D129–40. 10.1093/nar/gkab1030 PMC8728300 34850121

[27] Ampong I. Metabolic and Metabolomics Insights into Dilated Cardiomyopathy. Ann Nutr Metab. 2022;78(3):147–55. 10.1159/000524722 35472668

[28] Conte F, Sam JE, Lefeber DJ, Passier R. Metabolic Cardiomyopathies and Cardiac Defects in Inherited Disorders of Carbohydrate Metabolism: A Systematic Review. Int J Mol Sci. 2023;24(10):8632. 10.3390/ijms24108632 PMC10218694 37239976

[29] Alonso-Peña M, Espinosa-Escudero R, Herraez E, Briz O, Cagigal ML, Gonzalez-Santiago JM, et al. Beneficial effect of ursodeoxycholic acid in patients with acyl-CoA oxidase 2 (ACOX2) deficiency-associated hypertransaminasemia. Hepatology. 2022;76(5):1259–74. 10.1002/hep.32517 PMC9796151 35395098

[30] Desai MS, Mathur B, Eblimit Z, Vasquez H, Taegtmeyer H, Karpen SJ, et al. Bile acid excess induces cardiomyopathy and metabolic dysfunctions in the heart. Hepatology. 2017;65(1):189–201. 10.1002/hep.28890 PMC5299964 27774647

[31] Fang Y, Xu X, Ding J, Yang L, Doan MT, Karmaus PWF, Snyder NW, Zhao Y, Li JL, Li X. Histone crotonylation promotes mesoendodermal commitment of human embryonic stem cells. Cell Stem Cell. 2021;28(4):748–e7637. 10.1016/j.stem.2020.12.009 PMC8026719 33450185

[32] He A, Chen X, Tan M, Chen Y, Lu D, Zhang X, Dean JM, Razani B, Lodhi IJ. Acetyl-CoA Derived from Hepatic Peroxisomal β-Oxidation Inhibits Autophagy and Promotes Steatosis via mTORC1 Activation. Mol Cell. 2020;79(1):30–e424. 10.1016/j.molcel.2020.05.007 PMC7335356 32473093

[33] Ye S, Xu W, Chen Z, Zhu C, Ge Q, Lu J, et al. ACOX2 destabilizes the MRE11-RAD50-NBS1 complex and boosts anticancer immunity via the cGAS-STING pathway in clear cell renal cell carcinoma. Mol Cancer. 2025;24(1):263. 10.1186/s12943-025-02420-9 PMC12538886 41121283

[34] Liu X, He A, Lu D, Hu D, Tan M, Abere A, et al. Peroxisomal metabolism of branched fatty acids regulates energy homeostasis. Nature. 2025;646(8087):1223–31. 10.1038/s41586-025-09517-7 PMC12571886 40963015

[35] Li F, He X, Ye D, Lin Y, Yu H, Yao C, Huang L, Zhang J, Wang F, Xu S, Wu X, Liu L, Yang C, Shi J, He X, Liu J, Qu Y, Guo F, Zhao J, Xu W, Zhao S. NADP(+)-IDH Mutations Promote Hypersuccinylation that Impairs Mitochondria Respiration and Induces Apoptosis Resistance. Mol Cell. 2015;60(4):661–75. 10.1016/j.molcel.2015.10.017 26585387

[36] Gut P, Matilainen S, Meyer JG, Pällijeff P, Richard J, Carroll CJ, et al. SUCLA2 mutations cause global protein succinylation contributing to the pathomechanism of a hereditary mitochondrial disease. Nat Commun. 2020;11(1):5927. 10.1038/s41467-020-19743-4 PMC7684291 33230181

[37] Liu W, Wang X, Xu D, Gong F, Pei L, Yang S, et al. SIRT5 mediated succinylation of SUCLA2 regulates TCA cycle dysfunction and mitochondrial damage in pancreatic acinar cells in acute pancreatitis. Biochim Biophys Acta Mol Basis Dis. 2025;1871(3):167613. 10.1016/j.bbadis.2024.167613 39643219

[38] Lu X, Yang P, Zhao X, Jiang M, Hu S, Ouyang Y, Zeng L, Wu J. OGDH mediates the inhibition of SIRT5 on cell proliferation and migration of gastric cancer. Exp Cell Res. 2019;382(2):111483. 10.1016/j.yexcr.2019.06.028 31247190

