# Supplementary material for: Acox2 deficiency induces cardiac dysfunction via cross-organ metabolic reprogramming in mice
Source: J Transl Med. 2026 Aug 10;24:1401. doi: 10.1186/s12967-026-08797-5 (PMC13741627; doi:10.1186/s12967-026-08797-5)
Supplement: Supplementary file 1 [file 12967_2026_8797_MOESM3_ESM.pdf]

Supplementary Figure Legends

Figure S1. Acox2 expression in the mouse heart throughout the lifespan. According to the Expression Atlas database, Acox2 expression is consistently reported as below the detection threshold. Representative data from embryonic day 12.5 (E12.5) to postnatal week 8 (P8) are displayed as indicated by the reference: https://www.informatics.jax.org/gxd/summary?markerMgiId=MGI:1934852&structureID#gxd

Figure S2. Histological and fluorescence staining of mouse kidney and heart. (A) H&E staining of kidney tissue in Acox2 KO and WT mice showing no obvious histological abnormalities in the renal cortex or medulla. (B–C) Serum blood urea nitrogen (BUN) (B) and creatinine (CREA) (C) levels in Acox2 KO mice compared with WT controls at 4 months of age. (D) TUNEL staining of heart samples from Acox2 KO and WT mice.

Figure S3. Antibody screening for four classical PTMs. (A–D) Representative images of succinylation (A), acetylation (B), crotonylation (C), and ubiquitination (D) blots with long exposure times. Full-length Coomassie blue staining of each gel served as the loading control (left for A–C; bottom for E). (F) Four visible bands (#1–4) of ubiquitinated proteins were quantified to assess potential changes. Data are presented as the mean ± SEM; p-values were determined by a two-tailed Student's t-test ($n = 3$). ns, not significant.

Figure S4. MS/MS spectra of the succinylation sites: K86 in Tnnc1, K83 and K435 in Myh6, and K256 in Tln.

Figure S5. Validation of Flag-tagged Myl3, Vcl, and Tnnc1 recombinant protein expression in HEK-293T cells via immunoprecipitation assays.

Figure S6. Succinic acid levels in Acox2 KO mouse hearts. (A) Representative mass spectrometry peaks of succinic acid standards at a concentration of 1 μM. (B–C) Representative mass spectrometry peaks of succinic acid intensities in heart samples from Acox2 KO and WT mice. (D–E) Quantification of relative succinic acid levels in the heart and serum of Acox2 KO and WT mice. Data are presented as the mean ± SEM ($n = 5$ mice). Statistical significance was determined using a two-tailed Student's t-test; ns, not significant. (F–G) Representative mass spectrometry peaks of succinyl-CoA standards at 10 μM and 1 mM.

Table S1. List of 52 downregulated succinylation sites on myofibrillar and mitochondrial proteins in Acox2 KO mouse hearts.

Fig.S1

Genes (1) | Assays (10) | Assay results (53) | Images (0) | Tissue x Stage Matrix | Tissue x Gene Matrix | Heat Map

Export: Text File   Show Additional Sample Data

Gene	Result Details	Assay Type	Age	Structure	Cell Type	Sex	Detected?	TPM Level (RNA-Seq)	Images	Mutant Allele(s)	Reference
Acox2	data (E-MTAB-6798)	RNA-Seq	E12.5	TS20: heart		Female	No	Below Cutoff			E-MTAB-6798 Mouse RNA-seq time-series of the development of seven major organs
Acox2	data (E-MTAB-6798)	RNA-Seq	E12.5	TS20: heart		Male	No	Below Cutoff			E-MTAB-6798 Mouse RNA-seq time-series of the development of seven major organs
Acox2	data (E-MTAB-6798)	RNA-Seq	E13.5	TS21: heart		Female	No	Below Cutoff			E-MTAB-6798 Mouse RNA-seq time-series of the development of seven major organs
Acox2	data (E-MTAB-6798)	RNA-Seq	E13.5	TS21: heart		Male	No	Below Cutoff			E-MTAB-6798 Mouse RNA-seq time-series of the development of seven major organs
Acox2	data (E-MTAB-6798)	RNA-Seq	E14.5	TS22: heart		Female	No	Below Cutoff			E-MTAB-6798 Mouse RNA-seq time-series of the development of seven major organs
Acox2	data (E-MTAB-6798)	RNA-Seq	E14.5	TS22: heart		Male	No	Below Cutoff			E-MTAB-6798 Mouse RNA-seq time-series of the development of seven major organs
Acox2	data (E-MTAB-6798)	RNA-Seq	E15.5	TS23: heart		Female	No	Below Cutoff			E-MTAB-6798 Mouse RNA-seq time-series of the development of seven major organs
Acox2	data (E-MTAB-6798)	RNA-Seq	E15.5	TS23: heart		Male	No	Below Cutoff			E-MTAB-6798 Mouse RNA-seq time-series of the development of seven major organs
Acox2	data (E-MTAB-6798)	RNA-Seq	E16.5	TS24: heart		Male	No	Below Cutoff			E-MTAB-6798 Mouse RNA-seq time-series of the development of seven major organs
Acox2	data (E-MTAB-6798)	RNA-Seq	E16.5	TS24: heart		Female	No	Below Cutoff			E-MTAB-6798 Mouse RNA-seq time-series of the development of seven major organs
Acox2	data (E-MTAB-6798)	RNA-Seq	E17.5	TS25: heart		Male	No	Below Cutoff			E-MTAB-6798 Mouse RNA-seq time-series of the development of seven major organs
Acox2	data (E-MTAB-6798)	RNA-Seq	E17.5	TS25: heart		Female	No	Below Cutoff			E-MTAB-6798 Mouse RNA-seq time-series of the development of seven major organs
Acox2	data (E-MTAB-6798)	RNA-Seq	E18.5	TS26: heart		Female	No	Below Cutoff			E-MTAB-6798 Mouse RNA-seq time-series of the development of seven major organs
Acox2	data (E-MTAB-6798)	RNA-Seq	E18.5	TS26: heart		Male	No	Below Cutoff			E-MTAB-6798 Mouse RNA-seq time-series of the development of seven major organs
Acox2	data (E-MTAB-5449)	RNA-Seq	E18.5	TS26: heart left ventricle			No	Below Cutoff			E-MTAB-5449 Transcription profiling by RNA-seq of murine hearts at different developmental stages and adult mice with ischemia/reperfusion injury
Acox2	data (E-MTAB-6798)	RNA-Seq	P0	TS27: heart		Female	No	Below Cutoff			E-MTAB-6798 Mouse RNA-seq time-series of the development of seven major organs
Acox2	data (E-MTAB-6798)	RNA-Seq	P0	TS27: heart		Male	No	Below Cutoff			E-MTAB-6798 Mouse RNA-seq time-series of the development of seven major organs
Acox2	data (E-MTAB-5449)	RNA-Seq	P2	TS27: heart left ventricle			No	Below Cutoff			E-MTAB-5449 Transcription profiling by RNA-seq of murine hearts at different developmental stages and adult mice with ischemia/reperfusion injury
Acox2	data (E-MTAB-6798)	RNA-Seq	P3	TS27: heart		Female	No	Below Cutoff			E-MTAB-6798 Mouse RNA-seq time-series of the development of seven major organs
Acox2	data (E-MTAB-6798)	RNA-Seq	P3	TS27: heart		Male	No	Below Cutoff			E-MTAB-6798 Mouse RNA-seq time-series of the development of seven major organs
Acox2	data (E-MTAB-6798)	RNA-Seq	P14	TS28: heart		Female	No	Below Cutoff			E-MTAB-6798 Mouse RNA-seq time-series of the development of seven major organs
Acox2	data (E-MTAB-6798)	RNA-Seq	P14	TS28: heart		Male	No	Below Cutoff			E-MTAB-6798 Mouse RNA-seq time-series of the development of seven major organs
Acox2	data (E-MTAB-599)	RNA-Seq	P adult	TS28: heart			No	Below Cutoff			E-MTAB-599 RNA-seq of mouse DBA/2J x C57BL/6J heart, hippocampus, liver, lung, spleen and thymus
Acox2	data (E-MTAB-2801)	RNA-Seq	P adult	TS28: heart left ventricle		Male	No	Below Cutoff			E-MTAB-2801 Strand-specific RNA-seq of nine mouse tissues
Acox2	data (E-MTAB-2801)	RNA-Seq	P adult	TS28: heart left ventricle		Male	No	Below Cutoff			E-MTAB-2801 Strand-specific RNA-seq of nine mouse tissues
Acox2	data (E-GEOD-72165)	RNA-Seq	P adult	TS28: carotid body			No	Below Cutoff			E-GEOD-72165 Expression profile of mouse carotid body and adrenal medulla [RNA-Seq]
Acox2	data (E-MTAB-6798)	RNA-Seq	P28	TS28: heart		Male	No	Below Cutoff			E-MTAB-6798 Mouse RNA-seq time-series of the development of seven major organs
Acox2	data (E-MTAB-6798)	RNA-Seq	P28	TS28: heart		Female	No	Below Cutoff			E-MTAB-6798 Mouse RNA-seq time-series of the development of seven major organs
Acox2	data (E-MTAB-5449)	RNA-Seq	P w 4	TS28: heart left ventricle			No	Below Cutoff			E-MTAB-5449 Transcription profiling by RNA-seq of murine hearts at different developmental stages and adult mice with ischemia/reperfusion injury
Acox2	data (E-GEOD-74747)	RNA-Seq	P w 8	TS28: heart		Male	Yes	Low			E-GEOD-74747 RNA-seq of 9 tissues from an adult male C57BL/6 mouse
Acox2	data (E-MTAB-9361)	RNA-Seq	P w 8	TS28: heart left ventricle		Male	No	Below Cutoff		$Ttn^{tm1Mgot}/Ttn^{tm1Mgot}$	E-MTAB-9361 Heart RNA-seq of therapeutic RBM20 antisense oligonucleotide (ASO) treatment in a mouse model of heart failure with preserved ejection fraction (HFpEF)
Acox2	data (E-MTAB-9361)	RNA-Seq	P w 8	TS28: heart left ventricle		Male	No	Below Cutoff			E-MTAB-9361 Heart RNA-seq of therapeutic RBM20 antisense oligonucleotide (ASO) treatment in a mouse model of heart failure with preserved ejection fraction (HFpEF)
Acox2	data (E-MTAB-6798)	RNA-Seq	P63	TS28: heart		Male	No	Below Cutoff			E-MTAB-6798 Mouse RNA-seq time-series of the development of seven major organs
Acox2	data (E-MTAB-6798)	RNA-Seq	P63	TS28: heart		Female	No	Below Cutoff			E-MTAB-6798 Mouse RNA-seq time-series of the development of seven major organs
Acox2	data (E-MTAB-3662)	RNA-Seq	P w 16	TS28: heart ventricle		Male	No	Below Cutoff		$Arl10^{tm1.1(KOMP)Vlcg}/Arl10^{tm1.1(KOMP)Vlcg}$	E-MTAB-3662 Pilot KOMP knockout mouse strains
Acox2	data (E-MTAB-3662)	RNA-Seq	P w 16	TS28: heart ventricle		Male	No	Below Cutoff			E-MTAB-3662 Pilot KOMP knockout mouse strains
Acox2	data (E-GEOD-65775)	RNA-Seq	P m 6	TS28: heart		Male	No	Below Cutoff		Htt^{tm1Mfc}/Htt^{+}	E-GEOD-65775 Transcriptome profiling in knock-in mouse models of Huntington's disease [AllelicSeries_6month_TissueSurvey_mRNA]
Acox2	data (E-GEOD-65775)	RNA-Seq	P m 6	TS28: heart		Male	No	Below Cutoff			E-GEOD-65775 Transcriptome profiling in knock-in mouse models of Huntington's disease [AllelicSeries_6month_TissueSurvey_mRNA]
Acox2	data (E-GEOD-65775)	RNA-Seq	P m 6	TS28: heart		Female	No	Below Cutoff		Htt^{tm1Mfc}/Htt^{+}	E-GEOD-65775 Transcriptome profiling in knock-in mouse models of Huntington's disease [AllelicSeries_6month_TissueSurvey_mRNA]
Acox2	data (E-GEOD-65775)	RNA-Seq	P m 6	TS28: heart		Female	No	Below Cutoff			E-GEOD-65775 Transcriptome profiling in knock-in mouse models of Huntington's disease [AllelicSeries_6month_TissueSurvey_mRNA]

Fig.S2

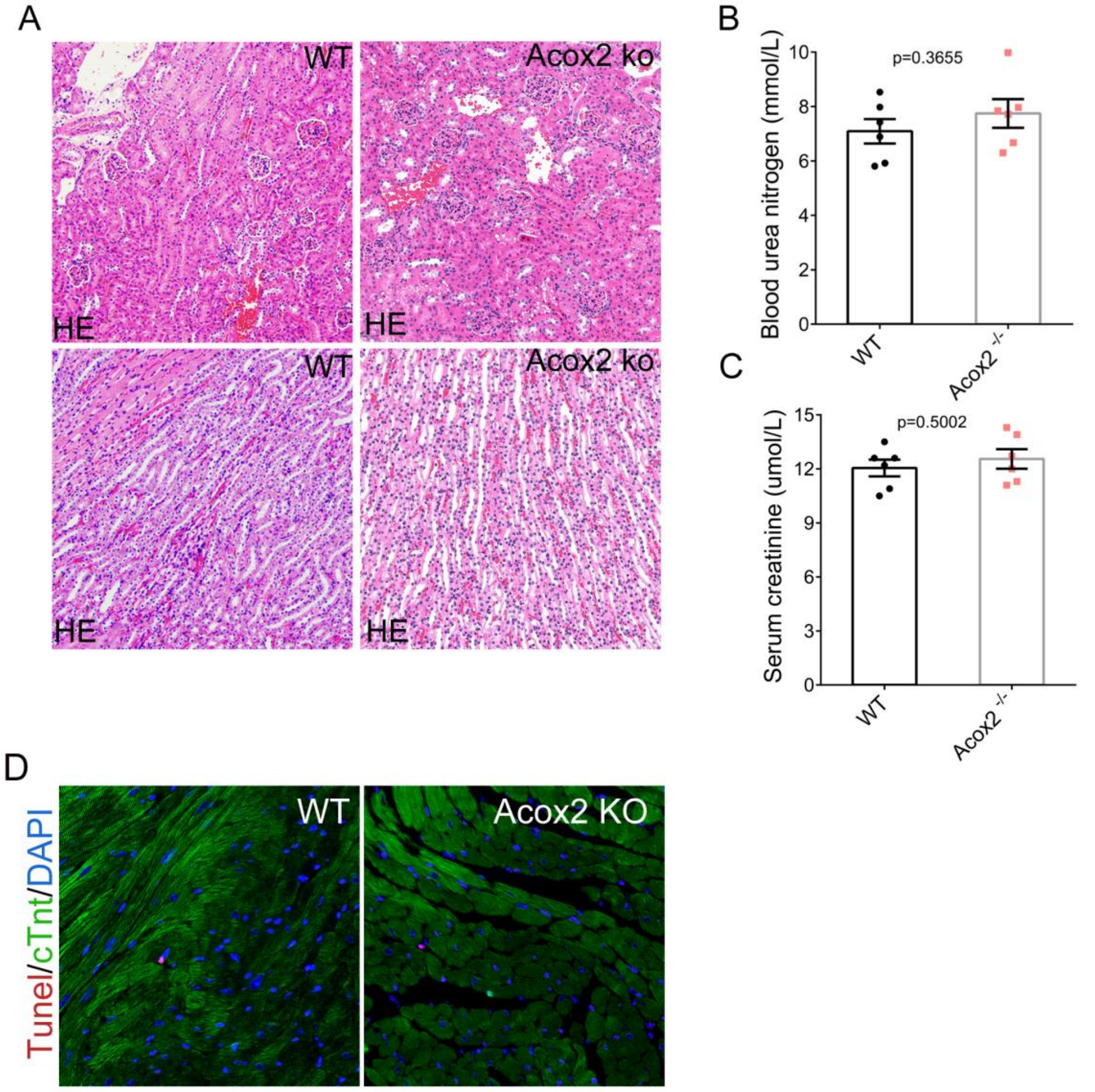

A
WT
Acox2 ko
HE
HE
WT
Acox2 ko
HE
HE
B
Blood urea nitrogen (mmol/L)
p=0.3655
10
8
6
4
2
0
WT
Acox2 -/-
C
Serum creatinine (umol/L)
p=0.5002
15
12
9
6
3
0
WT
Acox2 -/-
D
Tunel/cTnt/DAPI
WT
Acox2 KO

Fig.S3

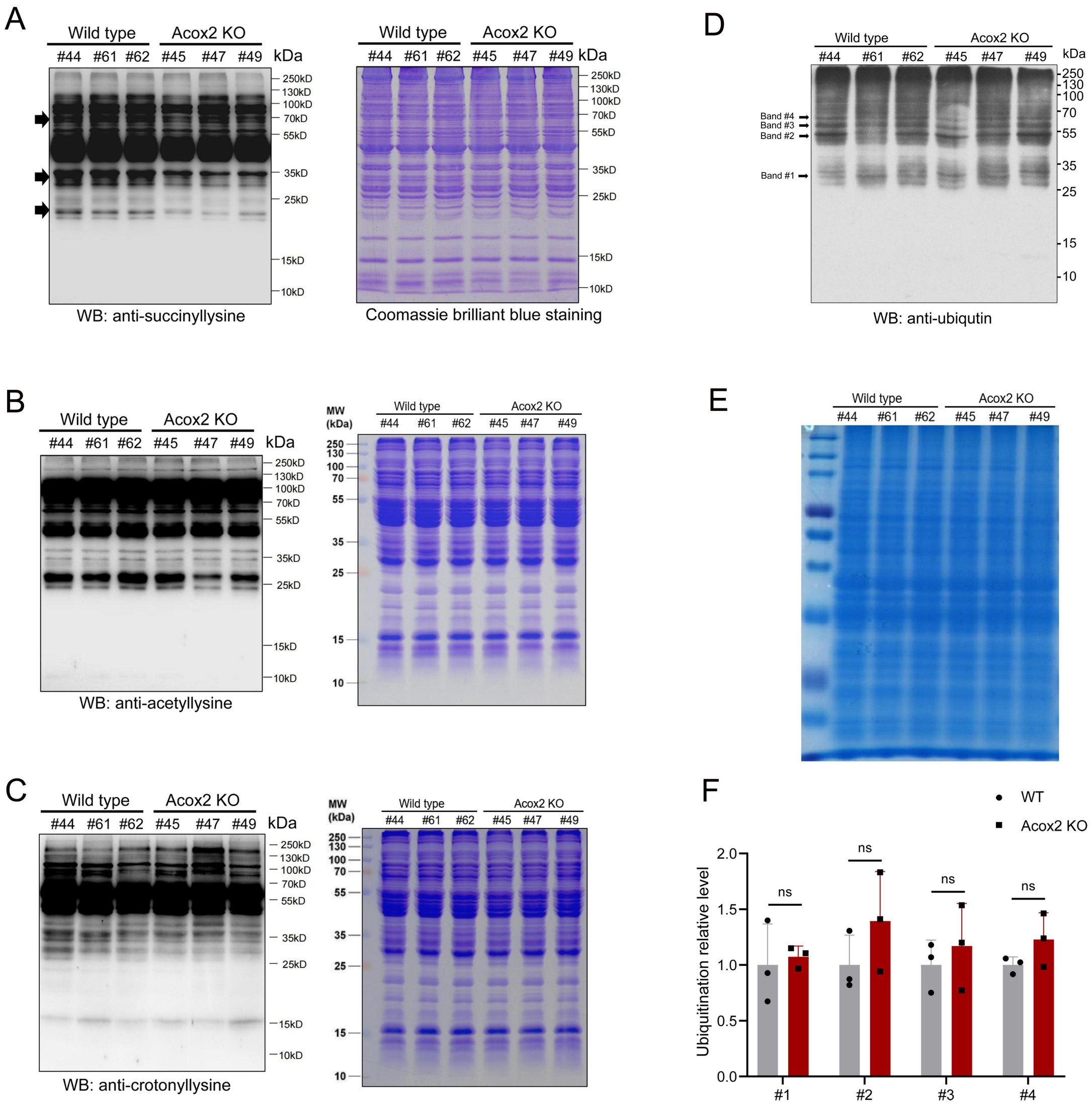
A
Wild type
Acox2 KO
#44 #61 #62 #45 #47 #49 kDa
250kD
130kD
100kD
70kD
55kD
35kD
25kD
15kD
10kD
WB: anti-succinyllysine
Coomassie brilliant blue staining
B
WB: anti-acetyllysine
MW (kDa)
250
130
100
70
55
35
25
15
10
C
WB: anti-crotonyllysine
D
Band #4
Band #3
Band #2
Band #1
WB: anti-ubiqutin
E
F
WT
Acox2 KO
Ubiquitination relative level
2.0
1.5
1.0
0.5
0.0
ns
#1
#2
#3
#4

Fig.S4

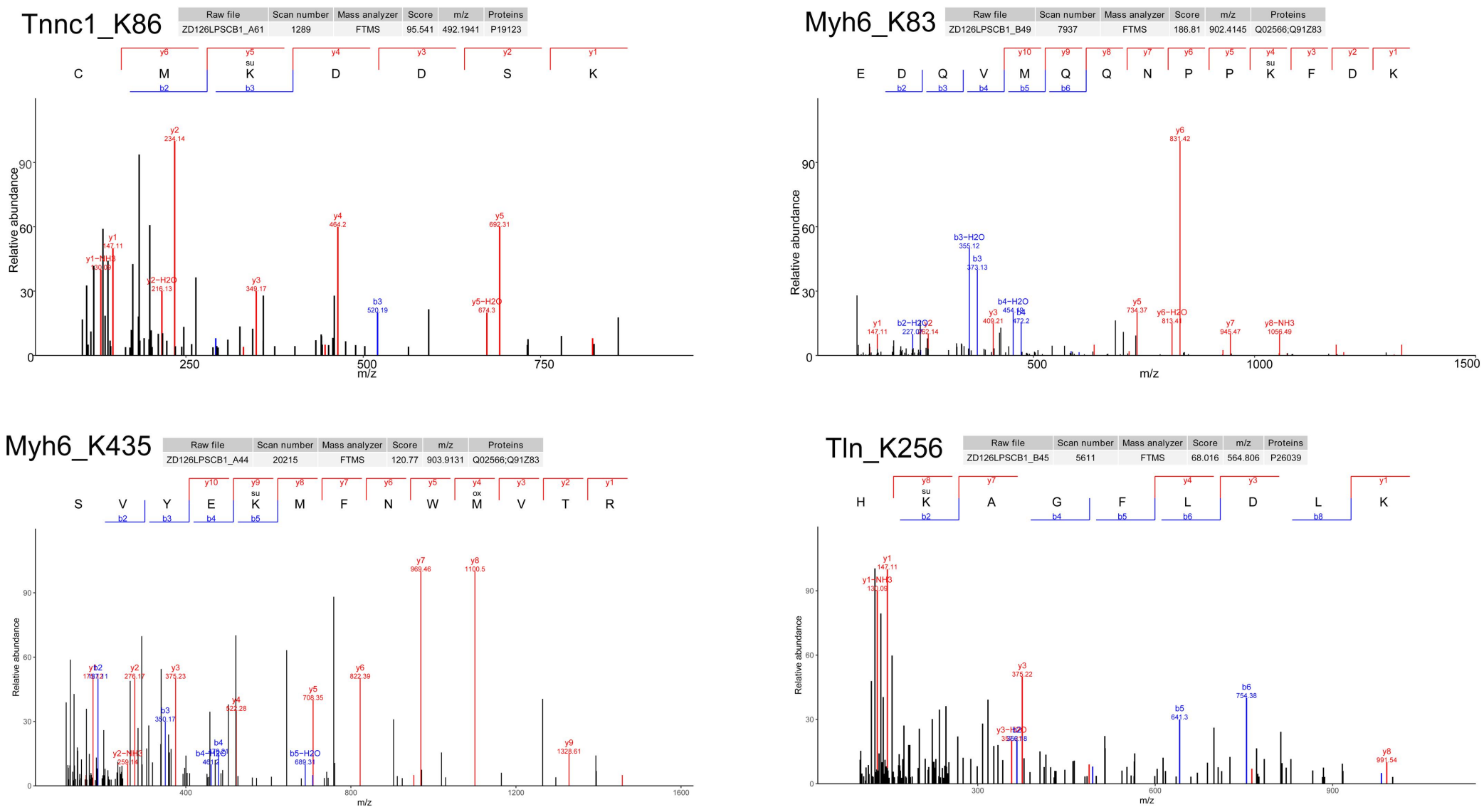

Tnnc1_K86
Raw file | Scan number | Mass analyzer | Score | m/z | Proteins
ZD126LPSCB1_A61 | 1289 | FTMS | 95.541 | 492.1941 | P19123
C M K D D S K
Relative abundance
m/z
Myh6_K83
Raw file | Scan number | Mass analyzer | Score | m/z | Proteins
ZD126LPSCB1_B49 | 7937 | FTMS | 186.81 | 902.4145 | Q02566;Q91Z83
E D Q V M Q Q N P P K F D K
Relative abundance
m/z
Myh6_K435
Raw file | Scan number | Mass analyzer | Score | m/z | Proteins
ZD126LPSCB1_A44 | 20215 | FTMS | 120.77 | 903.9131 | Q02566;Q91Z83
S V Y E K M F N W M V T R
Relative abundance
m/z
Tln_K256
Raw file | Scan number | Mass analyzer | Score | m/z | Proteins
ZD126LPSCB1_B45 | 5611 | FTMS | 68.016 | 564.806 | P26039
H K A G F L D L K
Relative abundance
m/z

Fig.S5

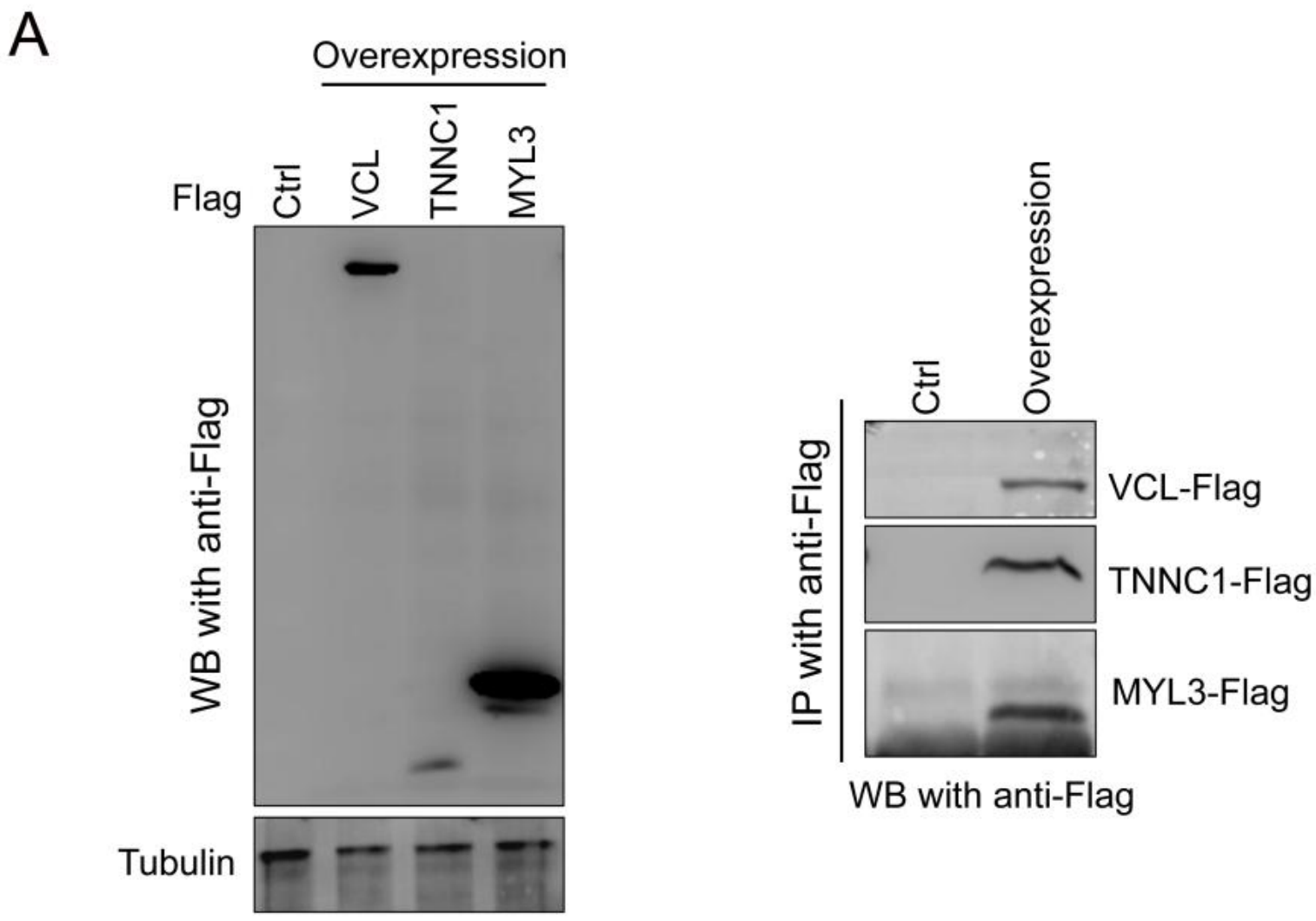
A
Overexpression
Flag
Ctrl
VCL
TNNC1
MYL3
WB with anti-Flag
Tubulin
Ctrl
Overexpression
IP with anti-Flag
VCL-Flag
TNNC1-Flag
MYL3-Flag
WB with anti-Flag

Fig.S6

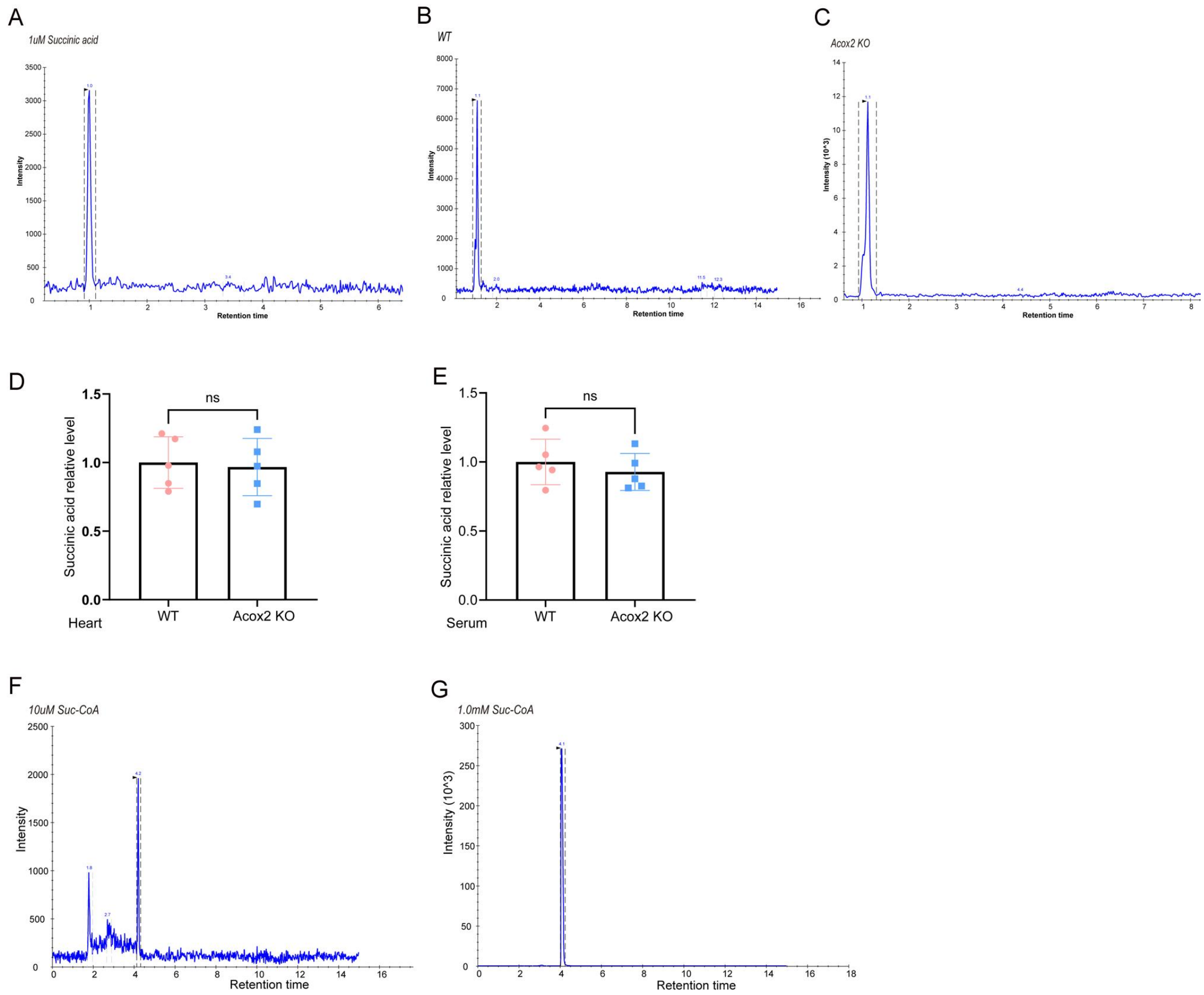

A
1uM Succinic acid
Intensity
Retention time
B
WT
Intensity
Retention time
C
Acox2 KO
Intensity (10^3)
Retention time
D
ns
Succinic acid relative level
WT
Acox2 KO
Heart
E
ns
Succinic acid relative level
WT
Acox2 KO
Serum
F
10uM Suc-CoA
Intensity
Retention time
G
1.0mM Suc-CoA
Intensity (10^3)
Retention time
