# Supplementary material for: Acox2 deficiency induces cardiac dysfunction via cross-organ metabolic reprogramming in mice
Source: J Transl Med. 2026 Aug 10;24:1401. doi: 10.1186/s12967-026-08797-5 (PMC13741627; doi:10.1186/s12967-026-08797-5)
Supplement: Supplementary file 2 [file 12967_2026_8797_MOESM4_ESM.pdf]

Genes		Position	Amino acid	Acox2 KO/WT Ratio	P value	Regulated
	Myofibril proteins					
1	Cryab	103	K	0.732	0.02050	Down
2	Des	240	K	0.665	0.00836	Down
3	Des	442	K	0.613	0.02650	Down
4	Dmd	2531	K	0.756	0.02529	Down
5	Hspb1	127	K	0.701	0.04796	Down
6	Myh6	435	K	0.584	0.03116	Down
7	Myh6	1559	K	0.728	0.04157	Down
8	Myh6	764	K	0.731	0.04740	Down
9	Myh6	83	K	0.421	0.02285	Down
10	Myh6	1244	K	0.729	0.04533	Down
11	Myh6	953	K	0.713	0.03555	Down
12	Myh7	83	K	0.483	0.04839	Down
13	Myl2	111	K	0.735	0.03125	Down
14	Myl3	109	K	0.683	0.03202	Down
15	Myl3	9	K	0.604	0.02977	Down
16	Myl3	4	K	0.629	0.00115	Down
17	Myom1	772	K	0.709	0.01711	Down
18	Myoz2	146	K	0.75	0.03350	Down
19	Nebl	143	K	0.688	0.01472	Down
20	Sptan1	1856	K	0.752	0.00015	Down
21	Sptan1	1334	K	0.7	0.00560	Down
22	Tnnc1	86	K	0.639	0.02798	Down
23	Tnni3	51	K	0.748	0.03831	Down
24	Ttn	4101	K	0.731	0.01549	Down
25	Ttn	22382	K	0.627	0.03687	Down
26	Ttn	34992	K	0.735	0.02276	Down
27	Ttn	27130	K	0.7	0.01688	Down
28	Ttn	19909	K	0.729	0.01487	Down
29	Ttn	27202	K	0.675	0.02754	Down
30	Vcl	453	K	0.51	0.02356	Down
	Mitochondria proteins					
1	Acadvl	508	K	0.713	0.04462	Down
2	Acat1	260	K	0.765	0.00717	Down
3	Atp5f1a	194	K	0.668	0.01329	Down
4	Atp5pd	32	K	0.746	0.02687	Down
5	Bdh1	258	K	0.582	0.03809	Down
6	Bdh1	280	K	0.731	0.00649	Down
7	Coq9	51	K	0.648	0.04141	Down
8	Cox6c	61	K	0.693	0.01940	Down
9	Cs	321	K	0.749	0.01495	Down
10	Fh	470	K	0.764	0.00461	Down
11	Gatd3a	217	K	0.61	0.04570	Down

12	Hadha	411	K	0.678	0.03314	Down
13	Hint2	128	K	0.764	0.03300	Down
14	Immt	352	K	0.759	0.01900	Down
15	Macrod1	146	K	0.76	0.00137	Down
16	Msra	179	K	0.746	0.01929	Down
17	Mtnd4	21	K	0.71	0.02373	Down
18	Ndufb11	43	K	0.616	0.00484	Down
19	Ogdh	999	K	0.7	0.00541	Down
20	Shmt2	469	K	0.756	0.04903	Down
21	Slc25a3	94	K	0.708	0.03923	Down
22	Sucla2	93	K	0.743	0.02785	Down
